# Global transcriptomic network analysis of the crosstalk between microbiota and cancer-related cells in the oral-gut-lung axis

**DOI:** 10.3389/fcimb.2024.1425388

**Published:** 2024-08-20

**Authors:** Beatriz Andrea Otálora-Otálora, César Payán-Gómez, Juan Javier López-Rivera, Natalia Belén Pedroza-Aconcha, Claudia Aristizábal-Guzmán, Mario Arturo Isaza-Ruget, Carlos Arturo Álvarez-Moreno

**Affiliations:** ^1^ Grupo de Investigación INPAC, Unidad de Investigación, Fundación Universitaria Sanitas, Bogotá, Colombia; ^2^ Dirección Académica, Universidad Nacional de Colombia, Sede de La Paz, La Paz, Colombia; ^3^ Grupo de Investigación INPAC, Specialized Laboratory, Clinica Universitaria Colombia, Clínica Colsanitas S.A., Bogotá, Colombia; ^4^ Keralty, Sanitas International Organization, Grupo de Investigación INPAC, Fundación Universitaria Sanitas, Bogotá, Colombia; ^5^ Infectious Diseases Department, Clinica Universitaria Colombia, Clínica Colsanitas S.A., Bogotá, Colombia

**Keywords:** transcription factors, transcriptional regulatory network, cancer-related cells membrane receptors, microbiota, hallmarks of cancer, oral-gut-lung axis, gut-lung cancer

## Abstract

**Background:**

The diagnosis and treatment of lung, colon, and gastric cancer through the histologic characteristics and genomic biomarkers have not had a strong impact on the mortality rates of the top three global causes of death by cancer.

**Methods:**

Twenty-five transcriptomic analyses (10 lung cancer, 10 gastric cancer, and 5 colon cancer datasets) followed our own bioinformatic pipeline based on the utilization of specialized libraries from the R language and DAVID´s gene enrichment analyses to identify a regulatory metafirm network of transcription factors and target genes common in every type of cancer, with experimental evidence that supports its relationship with the unlocking of cell phenotypic plasticity for the acquisition of the hallmarks of cancer during the tumoral process. The network’s regulatory functional and signaling pathways might depend on the constant crosstalk with the microbiome network established in the oral-gut-lung axis.

**Results:**

The global transcriptomic network analysis highlighted the impact of transcription factors (SOX4, TCF3, TEAD4, ETV4, and FOXM1) that might be related to stem cell programming and cancer progression through the regulation of the expression of genes, such as cancer-cell membrane receptors, that interact with several microorganisms, including human T-cell leukemia virus 1 (HTLV-1), the human papilloma virus (HPV), the Epstein–Barr virus (EBV), and SARS−CoV−2. These interactions can trigger the MAPK, non-canonical WNT, and IFN signaling pathways, which regulate key transcription factor overexpression during the establishment and progression of lung, colon, and gastric cancer, respectively, along with the formation of the microbiome network.

**Conclusion:**

The global transcriptomic network analysis highlights the important interaction between key transcription factors in lung, colon, and gastric cancer, which regulates the expression of cancer-cell membrane receptors for the interaction with the microbiome network during the tumorigenic process.

## 1 Introduction

Cancers are a leading cause of mortality, accounting for 10 million deaths worldwide every year. Most cancer deaths (75.1%) occur in low- and middle-income countries, where capital spending is lower at 49.5%. In high income countries like the US, where the cancer mortality rate is lower than the median, twice the amount of capital is spent on cancer care than in low-income countries, in an attempt to improve cancer outcomes (Chow et al., 2022). The estimated global economic cost of cancers between 2020 and 2050 is $25.2 trillion international dollars. The three cancers with the highest economic investment are tracheal, bronchus, and lung cancer (15.4%); colon and rectum cancer (10.9%); and breast cancer (7.7%) (Chen et al., 2023). Lung cancer has the second highest incidence (11.4% new cases) after breast cancer (11.7%) and is the leading cause of cancer-associated deaths worldwide (18%) (Siegel et al., 2023). Colon cancer has the third highest incidence (10%) and is the second leading cause of cancer-associated deaths worldwide (9.4%) (Siegel et al., 2023). Gastric cancer has the fifth highest incidence (5.6%), after breast, lung, colorectum and prostate cancer, and is the third leading cause of cancer-associated deaths worldwide (7.7%) (Siegel et al., 2023).

Approximately 80–85% of lung cancers are non-small cell lung cancer (NSCLC), which has three main subtypes: adenocarcinoma, squamous cell carcinoma, and large cell carcinoma. Approximately 10–15% of all lung cancers are small cell lung cancer (SCLC), and its main subtypes are small cell carcinoma and mixed small cell/large cell cancer or combined small cell lung cancer (Zhang et al., 2023). The regions with the highest risk of lung cancer are Polynesia, Micronesia, Eastern Asia, Europe, and Northern America (Siegel et al., 2023). The distinction between SCLC and NSCLC is a key parameter in the therapeutic management of lung cancer, selecting chemotherapy regimens in NSCLC patients lacking targetable EGFR and BRAF mutations, ALK and ROS1 rearrangements, and PD-L1 overexpression (Galli and Rossi, 2021). Targeted therapies based on druggable oncogenic molecular alterations and immunotherapy interfering with the PD1/PD-L1 checkpoint are used in lung cancer treatment (Salmani-Javan et al., 2024).

Colon cancer has three major histological types: adenocarcinoma, mucinous adenocarcinoma, and signet ring cell carcinoma (Wu et al., 2019). Adenocarcinoma originating from epithelial cells of the colorectal mucosa is the most frequent (>90%) type, and well differentiated tumors (>95%) are characterized by glandular formation (Rawla et al., 2019). Rare types include neuroendocrine, squamous cell, adenosquamous, spindle cell, and undifferentiated carcinomas (Fleming et al., 2012). The regions with the highest risk of colon cancer are Europe, Eastern Asia, and North and South America (Siegel et al., 2023). Adenocarcinoma patients have the best prognoses, whereas signet ring cell carcinoma patients have the poorest prognoses, and some patients with these different pathological types have no statistical difference at the early stages of the disease, suggesting that, right now, different treatment strategies specific for a particular histological subset should be applied only in the advanced stages of colon cancer (Ackermann et al., 2018). Currently, there are no established clinical guidelines for the treatment of the different pathological types, as previous studies on the prognostic effects have yielded conflicting results (Ackermann et al., 2018).

Gastric cancer classifications [intestinal type, diffuse type, and mixed type (Lauren, 1965) and the World Health Organization (mixed gastric mucinous adenocarcinoma) (Kushima, 2022)] have been used in clinicopathological diagnoses (Meng et al., 2022a). The well differentiated intestinal type is sporadic and highly associated with environmental factors, especially *Helicobacter pylori* infection (Takenaka et al., 2007). The diffuse type is undifferentiated and characterized by the loss of expression of the adhesion protein E-cadherin, which is related to tumor metastasis (Guilford et al., 1998). Gastric adenocarcinomas are the most frequent (>90%) and are classified as cardia and non-cardia based on their anatomic site, and the main subtypes are intestinal and diffuse (Yang et al., 2011). The regions with the highest risk of gastric cancer risk are East Asia, Eastern Europe, and Central and South America (Siegel et al., 2023). The incidence varies between regions, showing a high heterogeneity attributed to the commensal and infectious microbiota community, environmental factors, epigenetic programming, and genetic traits such as immune response receptors characteristic of every population ancestry (Otálora-Otálora et al., 2023b). Gastric cancer treatment includes chemotherapy, molecularly targeted therapies, and therapeutic agents such as immune checkpoint inhibitors (ICIs) selected for every patient as humanized monoclonal antibodies that target inhibitory receptors (CTLA-4, PD-1, LAG-3, TIM-3, and PD-L1) expressed on T lymphocytes, antigen presenting cells, and tumor cells, where they elicit an anti-tumor response by stimulating the immune system (Franzin et al., 2020); they are effective in the microsatellite instability and ICI subtypes but quite ineffective in the GS subtype (Kim et al., 2018).

The treatment of gut and lung cancer through histologic characteristics is limited by the high tumor morphological heterogeneity making every case extremely unique, and the identification of genomic biomarkers (Otálora-Otálora et al., 2023b) is still limited by the small number of patients with a specific variation, thus this therapeutic approach has not significantly impacted the mortality rates of the top three global causes of cancer deaths (Siegel et al., 2023). The identification of targetable oncogenic drivers in solid tumor and liquid biopsy using high-throughput deep sequencing methods can change significantly the paradigm of histology determination as one of the mandatory steps in future therapeutic strategies, and genomics as the only sequencing technology involved in the development of treatments against cancer (Galli and Rossi, 2021). We have developed a bioinformatics pipeline that is capable of identifying the most frequently differentially expressed genes (DEGs) and transcription factors (TFs) during the establishment and progression of every type of tumor pathology as key biomarkers due to their association with the regulation of biological processes and signaling pathways related to the acquisition of the hallmarks of cancer (Otálora-Otálora et al., 2023a). We address the great complexity of cancer in a holistic way from the transcriptome. The transcriptome reflects the genetics, epigenetics, and microenvironment of tumor cells that largely determine their phenotype. The identification of co-expressed common and unique DEGs between cancer types and related inflammatory diseases (Otálora-Otálora et al., 2019, Otálora-Otálora et al., 2022a, Otálora-Otálora et al., 2022b, Otálora-Otálora et al., 2023a) helps to identify the following: gene regulatory networks (GRNs), which contain promising targets for the pharmaceutical treatment of cancer (Zhou et al., 2019); co-regulatory networks (CRNs), which contain protein-protein interaction complexes of TFs regulating transcription at every stage of the tumorigenic process (Otálora-Otálora et al., 2023a); and transcriptional regulatory networks (TRNs), which represent the group of TFs that can regulate the all-important DEGs for the acquisition of the hallmarks of cancer (Otálora-Otálora et al., 2023b).

**Figure 1:**
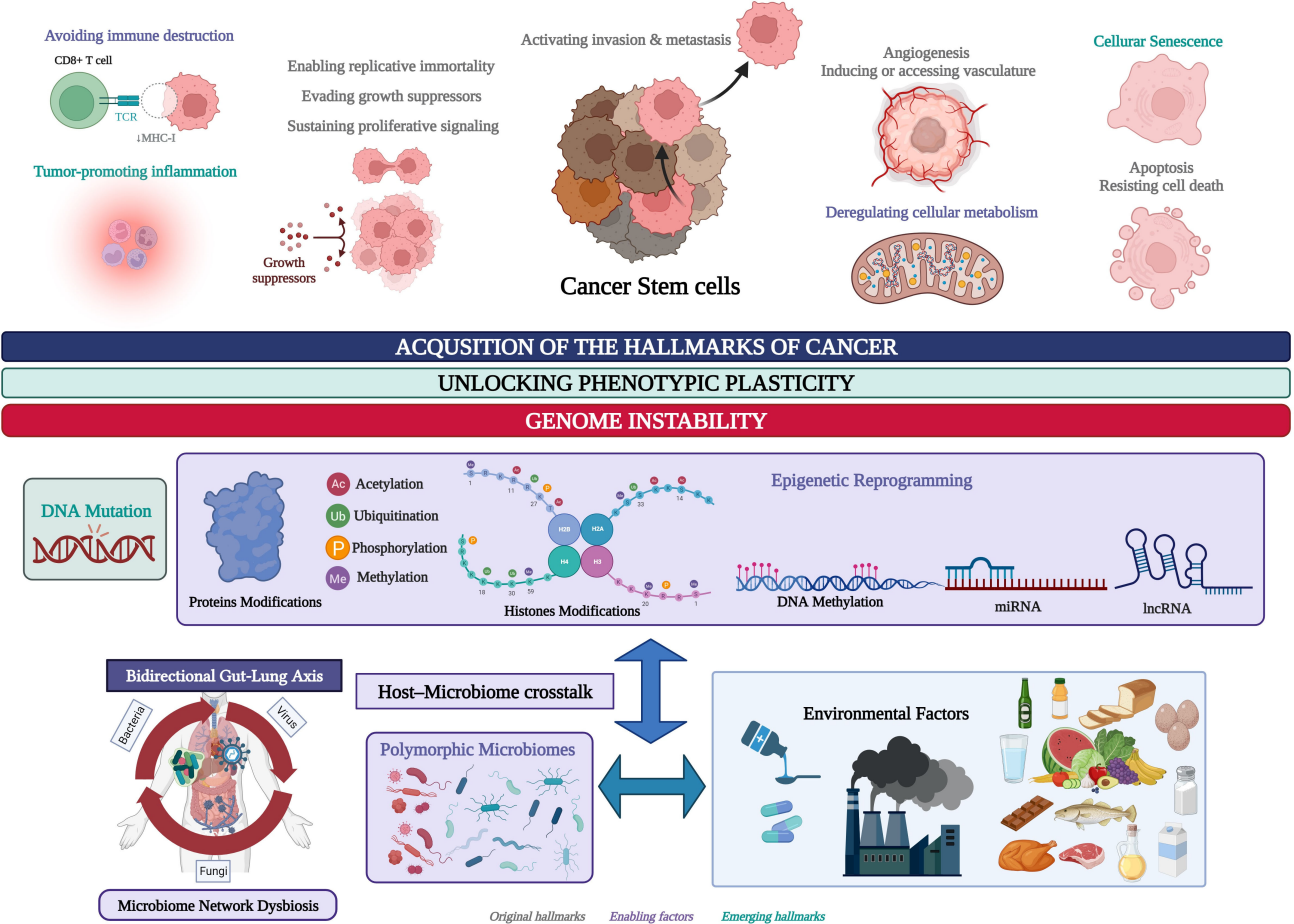
Environmental factors and the genomic and epigenomic regulatory processes involved in the unlocking of the phenotypic plasticity in cancer stem cells (CSC) and the acquisition of the hallmarks of cancer. Created with BioRender.com.

The polymorphic microbiome is a new dimension included in the hallmarks of cancer, along with epigenetic reprogramming, and both are considered constitute distinctive enabling characteristics that facilitate the acquisition of the hallmarks of cancer (**Figure 1**) (Hanahan, 2022). Our bioinformatic pipeline has evolved to identify a transcriptional regulatory metafirm of gut and lung cancer, the regulatory function of which might depend on the constant crosstalk with the microbiome network established in the gut-lung axis (Otálora-Otálora et al., 2023b). In this study, we aim to achieve the following: 1) identify the common DEGs in gut and lung cancers, highlighting the *membrane receptors* that are key for the communication between the host cells and the microbiota, and the downstream activation of signaling pathways involved in the regulation of gene expression, particularly the expression of key TFs that control the expression of the other DEGs in every type of cancer; 2) identify the regulatory interactions between the key TFs in a TRN as functional blocks of genes co-expressed for the control of gene expression and the acquisition of the hallmarks of cancer, and analyze its regulatory function over the other DEGs; and 3) perform a *global transcriptomic network analysis* combining the results of gene ontology and network bioinformatic analysis, along with a deep review of the scientific data, which ultimately might highlight the importance of the microbiota in the regulation of genomic (DNA mutations), transcriptomic (RNA expression), epigenomic, (DNA methylation, non-coding RNAs, histone, and protein modifications), proteomic, and metabolomic process stability through the establishment of a specific TRN in cancer-related cells, which in turn may control the interaction with the microbiome network to unlock phenotypic plasticity and acquire the hallmarks of cancer (**Figure 1**). Therefore, the highlighted TFs in a cancer TRN that could communicate with the microbiome network through specific membrane receptors may become interesting candidates that could be used as multiomic biomarkers for the development of specific diagnostic tools and treatments against cancer.

## 2 Materials and methods

### 2.1 Data selection, quality control, and construction of gene expression matrices

An advanced search was conducted on the National Center for Biotechnology Information (NCBI) GEO database (http://www.ncbi.nlm.nih.gov/geo/) to identify studies analyzing global gene expression in lung cancer, colon cancer, and gastric cancer. The search tool was used with the keywords “lung cancer,” “colon cancer,” and “gastric cancer”. Studies were limited to *Homo sapiens* as the organism, with expression profiling by array or RNA sequencing as the dataset type. The inclusion criteria encompassed studies that: (1) utilized any version of microarrays or any chemical of next generation sequencing; (2) analyzed at least three samples of each cancer type and at least three normal tissue samples for comparison; (3) provided raw data availability; and (4) passed quality control measures. All datasets underwent a quality control (QC) process to ensure their suitability for analysis. The initial step involved assessing signal comparability across different samples in each dataset to verify technical consistency. We then conducted a thorough examination of transcriptome correlations between samples to identify any significant deviations that might indicate poor quality samples. Principal component analysis (PCA) was employed as an additional tool for detecting low-quality samples. PCA helps visualize the variance within the dataset and allows for the identification of samples that significantly deviate from the main group. Samples exhibiting extreme variance or those that were outliers in the PCA plot were flagged as low quality. These low-quality samples, along with any datasets that did not meet our quality standards, were subsequently excluded from further analysis. Additionally, after processing and cleaning the datasets, we conducted an over-representation pathway analysis using the DAVID (Database for Annotation, Visualization, and Integrated Discovery) tool. This analysis was performed on the lists of DEGs obtained from each dataset individually. The aim was to confirm that the genes identified as differentially expressed were associated with known biological pathways relevant to cancer. This additional validation provided an extra layer of confidence in our results, ensuring that the selected genes were not only significantly different in expression but also implicated in critical biological processes related to the disease.

Of the total number of datasets analyzed, ten lung cancer datasets, five colon cancer datasets, and ten gastric cancer datasets (**Table 1**), passed the quality control phase and were used to obtain a gene expression matrix with Limma library in R (Ritchie et al., 2015), or DESeq2 (Love et al., 2014). For Affymetrix data, processing was conducted using the Limma R/Bioconductor software package (Ritchie et al., 2015). Data normalization and log2 transformation were carried out using the Robust Multi-array Average (RMA) algorithm. Owing to the presence of multiple probes for the same gene on Affymetrix chips, only the most informative probe, demonstrating the highest variability across experimental groups, was retained, whereas redundant probes were discarded. As noted in **Table 1**, some datasets have differences in the number of cases and controls. We have this in account by using Limma. Limma’s strength lies in its ability to fit linear models and adjust for varying group sizes. It employs an empirical Bayes approach that stabilizes the variance estimates by shrinking them toward a common value. This method enhances the accuracy of variance estimates, allowing Limma to efficiently handle datasets with unequal sample sizes. Consequently, Limma provides reliable and precise results even when there are significant differences in the sizes of the experimental groups. For RNA-Seq data analysis, preprocessing began directly with the gene count table available in GEO. Normalization of these count tables was performed using the method implemented in DESeq2 (Love et al., 2014) to address differences in library size and composition across samples. Subsequently, differential expression analysis was carried out using DESeq2 to identify genes exhibiting significant expression changes between experimental conditions. For both Affymetrix and RNA-Seq data, a gene was considered differentially expressed if the fold change in expression exceeded 1.5 or was less than −1.5, and the adjusted p-value was less than 0.05.

**Table 1:** Cancer datasets and number of samples. .

Cancer Datasets	Number of samples
GSE19804	Normal (60) vs. cancer NSCLC (60)
E-MTAB-3950	Normal (30) vs. early squamous lung carcinoma (30)
GSE108055	Normal (9) vs. small-cell lung cancer (54)
GSE10072	Normal (49) vs. lung adenocarcinoma (58)
GSE3268	Normal (5) vs. squamous lung cancer cells (5)
GSE52248	Normal (6) vs. lung adenocarcinoma (12)
GSE70089	Normal (3) vs. lung carcinoma (3)
GSE81089	Normal (19) vs. cancer NSCLC (199)
GSE84776	Normal (9) vs. squamous lung cancer (9)
E-MTAB-5231	Normal (18) vs. cancer NSCLC (22)
GSE9348	Normal (12) vs. colon cancer (70)
GSE23878	Normal (24) vs. colon cancer (35)
GSE24514	Normal (15) vs. colon cancer (34)
GSE35279	Normal (5) vs. colon cancer (74)
GSE4107	Normal (10) vs. colon cancer (12)
GSE113255	Normal gastric tissues (n = 10), gastric cancer diffuse mucinous (n = 10)
GSE113255	Normal gastric tissues (n = 10), gastric cancer diffuse differentiated (n = 42)
GSE63089	Normal gastric tissues (n = 45), gastric cancer (n = 45)
GSE54129	Normal gastric tissues (n = 21), gastric cancer (n = 111)
GSE33335	Normal gastric tissues (n = 25), gastric cancer (n = 25)
GSE26899	Normal gastric tissues (n = 12), gastric cancer intestinal (n = 8)
GSE26899	Normal gastric tissues (n = 12), gastric cancer diffuse (n = 7)
GSE13911	Normal gastric tissues (n = 31), gastric cancer (n = 38)
GSE13195	Normal gastric tissues (n = 25), gastric cancer (n = 23)
GSE2685	Normal gastric tissues (n = 8), gastric cancer (n = 22)

### 2.2 Gene functional annotation and gene networks analysis

The DEG lists of every dataset in every type of cancer were compared to identify the common overregulated and downregulated genes, highlighting membrane receptors, and TFs in at least seven datasets of lung and gastric cancer, and at least three datasets of colon cancer (**Supplementary Tables 1**-**3**). DAVID´s annotation tool was used to identify the related biological functions and signaling pathways associated with the common overregulated DEGs and deregulated TFs in every type of cancer (Sherman et al., 2022). The TRNs were constructed with the expression matrix of common TFs in every type of cancer in the Reconstruction of Transcriptional regulatory Networks and analysis of regulons (RTN) library (Groenevelt et al., 2024). The GRNs and CRNs were constructed with the expression matrix of common overexpressed DEGs, and deregulated TFs in every type of cancer with the CoRegNet library (Nicolle et al., 2015). The regulatory interactions between common deregulated TFs were analyzed based on TRNs and CRNs, and the regulators (TFs) of key receptors were identified through the analysis of the GRNs. All libraries were used under our own bioinformatic pipeline (**Supplementary Methods 2**).

### 2.3 Global transcriptomic network analysis of gut and lung cancer

Common DEGs of membrane receptors, TFs, and DEGs involved in signaling pathways associated with the interaction of cancer-related cells and microorganisms were highlighted by DAVID´s annotation analysis of all common DEGs in each type of cancer. The Results section focuses on the main TFs of every cancer type according to the TRN analysis and features their regulatory function according to the biological processes and signaling pathways that might control them during the tumorigenic process. The Discussion section considers the experimental evidence related to the TFs and signaling pathways involved in the host cell and microbiota interactions directly or through microbiota expressed proteins, with specific membrane receptors, identified as common DEGs in every cancer type. BioRender was used to show, in one image, all the knowledge generated by scientific research in the field of host-microbiome crosstalk, through cancer transcriptomic studies. Lung, colon, and gastric cancer TRNs represent intricate gene regulatory programs involved in tumorigenesis, whereas the co-regulatory analysis of TFs assesses the formation of key protein-protein interaction complexes that might be important for unlocking the phenotypic plasticity of cancer-related cells through the control of key regulatory programs in a specific spatial, temporal, and sequential manner, probably according to their communication with the characteristic microbiome network. Consequently, a global transcriptomic analysis was carried out based on a bioinformatic analysis to organize and present all the scientific evidence present in several publications in the field of microbiome and cancer, in an image. This image features the best known microorganisms and their proteins that interact with key membrane receptors deregulated in every cancer type, the actual evidence of the signaling pathways that they might activate, and the following control of gene expression programs through the activation of cancer TRNs that might be related to the unlocking of phenotypic plasticity and acquisition of the hallmarks of cancer, as well as the control of the gene expression of key TFs over the gene expression of the main membrane receptors that interact with the microbiome network, representing the impact of the TRN in the communication with the microbiome network during the tumorigenic process. In the Conclusion, we summarize for every cancer type, the key TFs and their regulatory interactions, the membrane receptors and the best-known microorganisms that interact with them, and the signaling pathways, with experimental evidence that might be used to establish the current state of microbiome and cancer research, which can be used as the guiding core and starting point for future multiomic studies in this field.

## 3 Results

### 3.1 Differentially expressed genes and transcription factors in tumorigenic processes of the gut-lung axis

**Figure 2:**
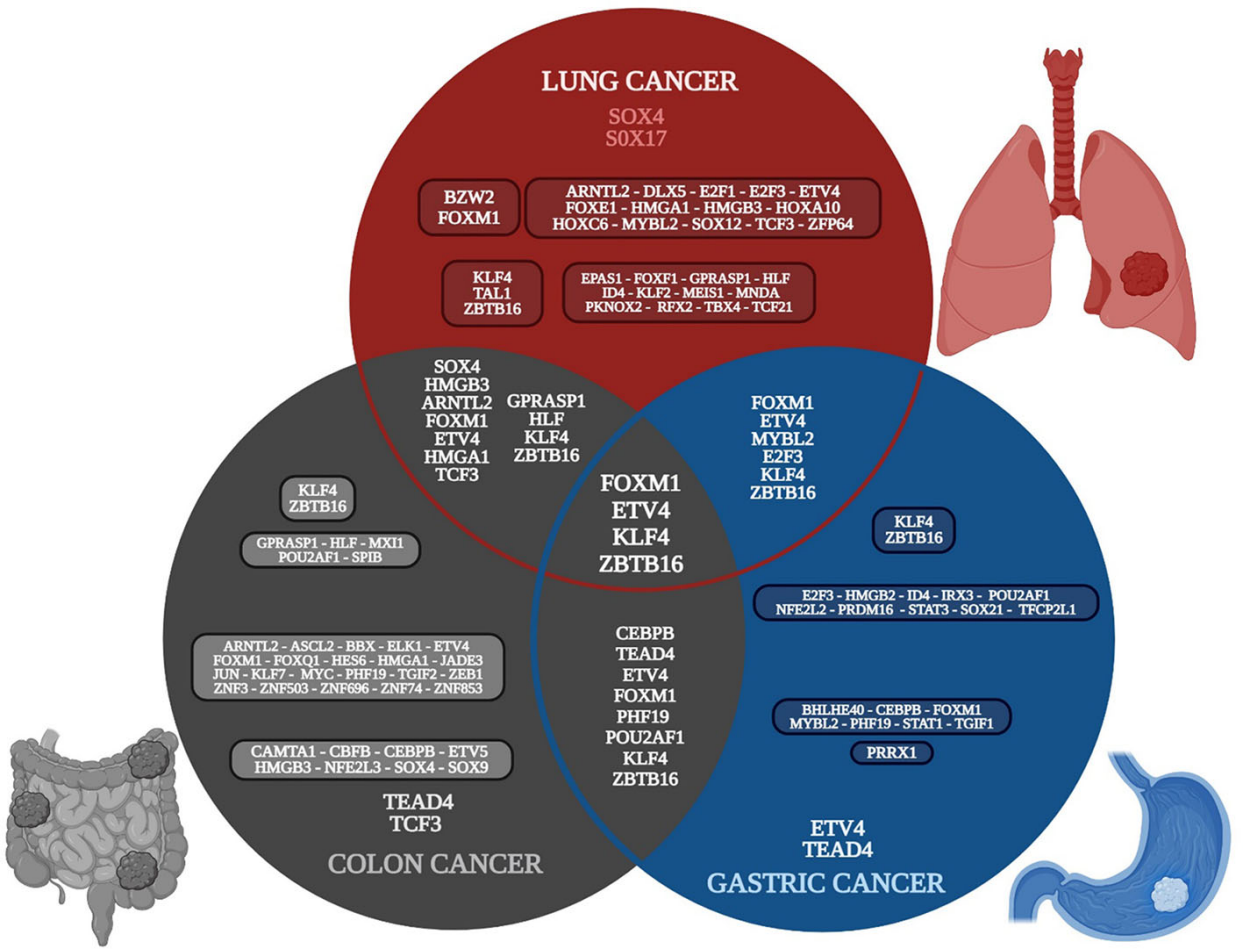
Venn diagram of the transcriptomic metafirm of gut-lung axis tumorigenic processes, with the common and unique deregulated transcription factors of each type of cancer. Created with BioRender.com.

Ten gene expression datasets of lung cancer were analyzed; 417 common overregulated genes and 438 downregulated genes were identified in at least seven of the datasets (**Supplementary Table 1**) (Otálora-Otálora et al., 2023a). Five gene expression datasets of colon cancer were analyzed; 474 common overregulated genes and 640 downregulated genes were identified in at least three of the datasets (**Supplementary Table 2**). Ten gene expression datasets of gastric cancer were analyzed; 195 common overregulated genes and 309 downregulated were identified in at least seven of the datasets (**Supplementary Table 3**). Eighteen overregulated and 16 downregulated TFs were identified in the lung cancer common genes list, 37 overregulated and seven downregulated TFs were identified in the colon cancer common DEGs list, and 10 overregulated and 12 downregulated TFs were identified in the gastric cancer common DEGs list. There were seven overregulated and four downregulated TFs in common between lung and colon cancer, three overregulated and three downregulated TFs in common between lung and gastric cancer, five overregulated and three downregulated TFs in common between colon and gastric cancer, and two overregulated and two downregulated TFs in common between the three types of cancer (**Figure 2**).

Lung cancer common overregulated DEGs are related to epigenetic reprogramming (phosphorylation, acetylation, methylation, hydroxylation, and ubiquitin-like conjugations [Ubls]), the formation of extracellular exosomes, cell division, apoptosis, adhesion, proliferation, and junctions, the regulation of transcription, the epigenetic regulation of gene expression, DNA damage, repair, recombination, and replication, the formation of TF complexes, the regulation of stem cell population maintenance, and signaling pathways involved in DNA replication, cellular senescence, base excision repair, viral carcinogenesis, the biosynthesis of co-factors, mismatch repair, the cell cycle, purines, and metabolism, Wnt, hepatitis B (HBV), Epstein–Barr (EBV), human papillomavirus (HPV), and human T-cell leukemia virus 1 infection (HTLV-I) (**Supplementary Table 1**). Colon cancer common overregulated DEGs are related to epigenetics reprogramming (phosphorylation, acetylation, methylation, and ubl conjugation), the formation of extracellular exosomes, cell adhesion, proliferation, apoptosis, junctions, migration, and division, the regulation of transcription, DNA damage, repair, and replication, the formation of TF complexes, and stem cell proliferation, as well as signaling involved in the cell cycle, proteoglycans in cancer, cellular senescence, amoebiasis, malaria, and hepatitis C virus (HCV), EBV, HPV, and HTLV-I infection (**Supplementary Table 2**). Gastric cancer overregulated common DEGs are related to epigenetic reprogramming (hydroxylation, phosphorylation, and ubl conjugation), the formation of extracellular exosomes, cell adhesion, proliferation, migration, division, apoptosis, junctions, and growth, DNA replication, angiogenesis, the regulation of telomerase activity, the regulation of the epithelial to mesenchymal transition (EMT), inflammatory response, angiogenesis, gene expression regulation, cellular response to TNF, innate immunity, and host-virus interaction, as well as signaling involved in malaria, amoebiasis, pertussis, Kaposi sarcoma-associated herpesvirus, and HPV (**Supplementary Table 3**).

### 3.2 Transcriptional and gene regulatory network analysis

#### 3.2.1 Lung cancer

**Figure 3:**
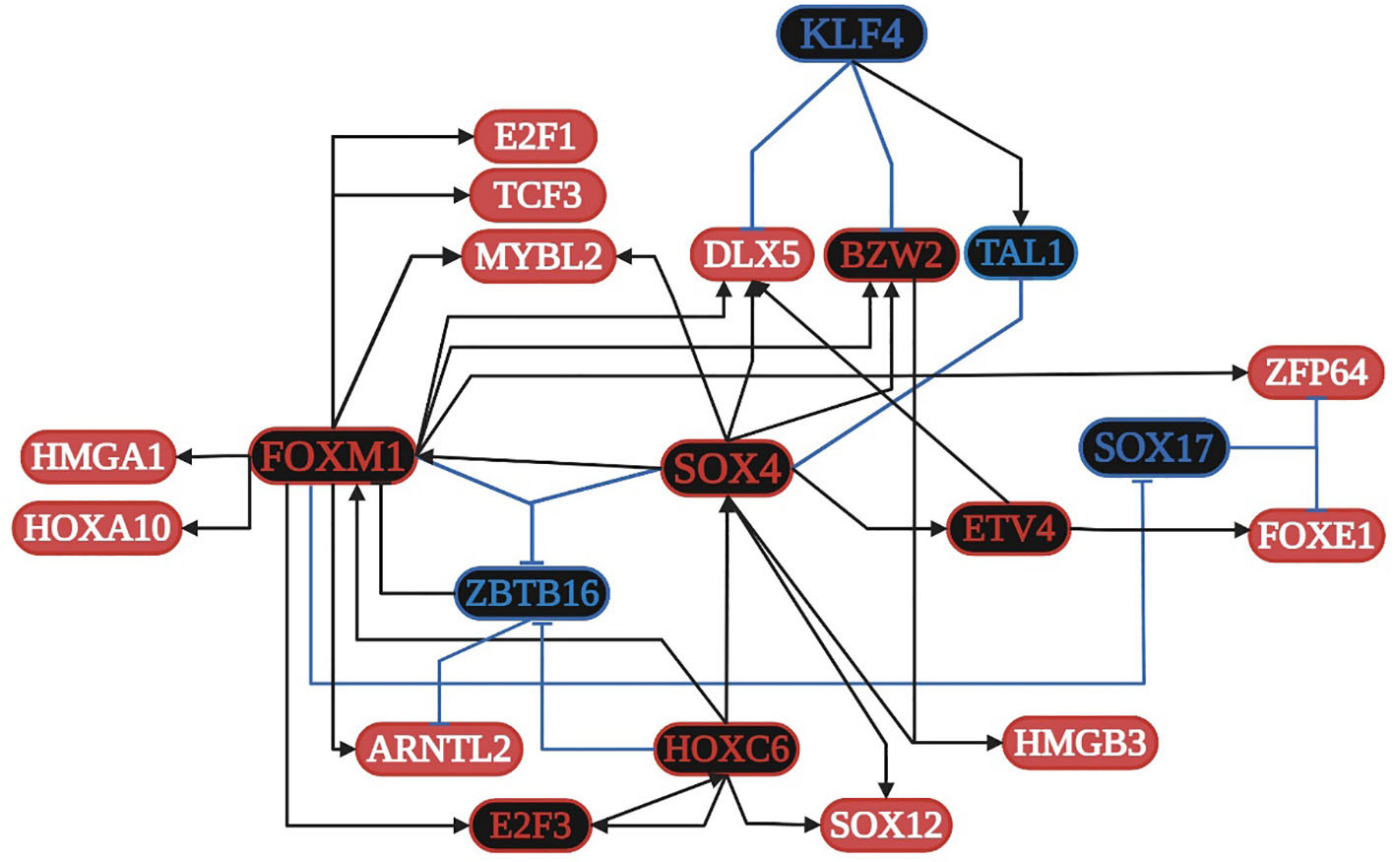
Transcriptional regulatory network (TRN) of common transcription factors (TFs) in lung cancer. Downregulated TFs are in blue, upregulated TFs are in red, and the key overregulated and downregulated TFs are in black. The black pointed arrows represent overregulation and the blue flat arrows represent downregulation. Created with BioRender.com.

SOX4 is overregulated in all ten lung cancer datasets and is related to: 1) apoptosis along with E2F1; 2) cell proliferation along with FOXM1, DLX5, and E2F3; 3) the formation of TF complexes along with E2F3, ARNTL2, HMGA1, HOXA10, and TCF3; 4) the positive regulation of transcription along with E2F1, FOXM1, ETV4, DLX5, FOXE1, HMGA1, and TCF3; and 5) the positive regulation of the Wnt signaling pathway along with DLX5 (**Supplementary Table 1**). FOXM1 is overregulated in nine lung cancer datasets and is related to: 1) the negative regulation of transcription along with E2F1, FOXE1, and HMGA1; 2) cellular senescence along with E2F1, E2F3, and MYBL2; and 3) DNA repair and damage. E2F1 is overregulated in seven lung cancer datasets and is related to: 1) HPV infection; 2) HBV and EBV virus infection along with E2F3; 3) the cell cycle and pathways in cancer along with E2F3; and 4) HTVL-I infection along with E2F3 and TCF3. According to the TRN of lung cancer (**Figure 3**), SOX4, FOXM1, ETV4, HOXC6, and E2F3 are the main positive regulators, whereas SOX17, KLF4, and ZBTB16 are the main negative regulators of transcription, controlling the expression of other TFs and target genes in lung cancer. Additionally, the TFs are capable of forming co-regulatory complexes in NSCLC (DLX5, BZW2, E2F3, FOXM1, HMGA1, HMGB3, HOXC6, MYBL2, and SOX4) and SCLC (DLX5, E2F1, E2F3, FOXM1, HOXC6, SOX4, and TCF3). According to DAVID analysis, SOX4 is regulated by acetylation, whereas DLX5 is regulated by phosphorylation; both positively regulate transcription and the canonical Wnt signaling pathway (**Supplementary Table 1**). SOX4 has 140 target genes in the GRN, which are in the nucleus, cytoplasm, cytoskeleton, and membrane, and are related to epigenetic reprogramming (phosphorylation and acetylation), host-virus interactions, five membrane receptors (HMMR, LRP8, PTPRF, SRPRB, and TNFRSF21), and signaling pathways related to HTLV-I and HBV infection. According to the TRN analysis, SOX4 can be regulated by HOXC6, whereas DLX5 can be regulated by SOX4, FOXM1, and ETV4 (**Figure 3**), and according to the co-regulatory network analysis, they both participate in the formation of TF complexes in both subtypes of lung cancer (NSCLC and SCLC) (**Supplementary Table 1**).

#### 3.2.2 Colon cancer

**Figure 4:**
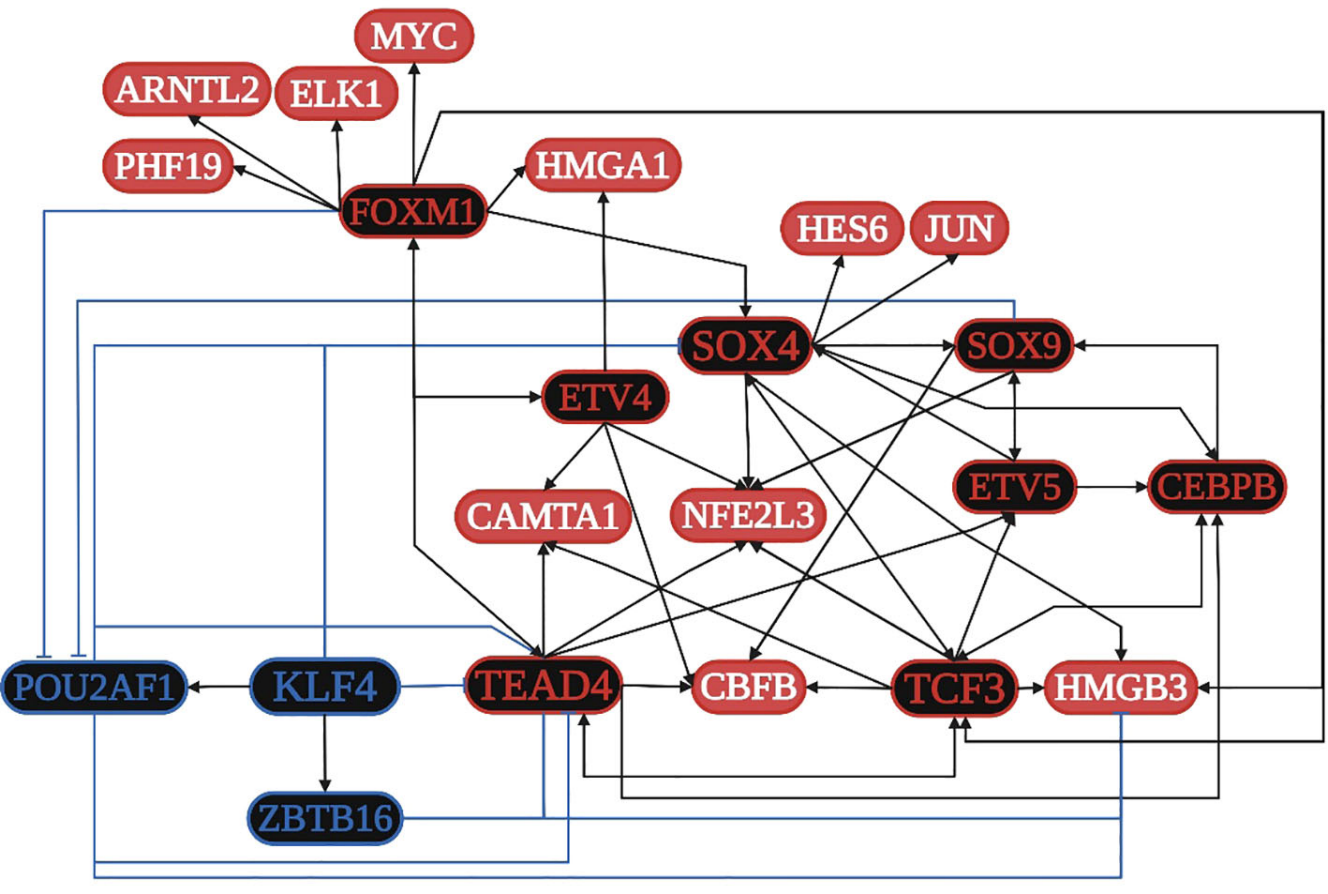
Transcriptional regulatory network (TRN) of common transcription factors (TFs) in colon cancer. Downregulated TFs are in blue, upregulated TFs are in red, and the key overregulated and downregulated TFs are in black. The black pointed arrows represent overregulation, and the blue flat arrows represent downregulation. Created with BioRender.com.

TCF3 is overregulated in four of the five datasets and is related to: 1) the positive regulation of transcription along with SOX4, SOX9, CEBPB, FOXM1, ETV4, JUN, ELK1, ARNTL2, and HMGA1; 2) the formation of a transcription regulatory complex along with TEAD4, JUN, SOX4, SOX9, ARNTL2, HES6, and HMGA1; 3) the positive regulation of transcription from the RNA polymerase II promoter along with TEAD4, ELK1, ETV4, ARNTL2, CAMTA1, HMGA1, CEBPB, ETV5, JUN, KLF7, MYC, SOX4, SOX9, ASCL2, CBFB, FOXM1, and ZEB1; 4) the negative regulation of transcription from the RNA polymerase II promoter along with CEBPB, ETV5, JUN, KLF7, MYC, NFE2L3, PHF19, SOX4, SOX9, ASCL2, CBFB, FOXM1, FOXQ1, HES6, TCFL5, ZEB1, and ZNF3; and 5) HTLV-I infection along with ELK1, JUN, and MYC. SOX4 is overregulated in four of the five datasets and is related to: 1) the positive regulation of proliferation along with SOX9, FOXM1, and MYC; 2) the regulation of apoptosis along with JUN, SOX9, and MYC; 3) the regulation of translation along with MYC; and 4) the regulation of the Wnt signaling pathway along with SOX9. JUN is overregulated in three of the five datasets and is related to: 1) colon cancer signaling pathways along with MYC; 2) the focal adhesion pathway along with ELK1; 3) pathways in cancer along with ELK1 and MYC; 4) EBV infection along with MYC; and 5) angiogenesis. MYC is overregulated in three of the five datasets and is related to: 1) cellular senescence along with FOXM1; 2) proteoglycans in cancer along with ELK1; and 3) HCV infection (**Supplementary Table 2**). According to the TRN analysis (**Figure 4**), TCF3 is related to the upregulation of TEAD4, SOX4, ETV5, CEBPB, CBFB, HMGB3, and NFE2L3; TEAD4 is related to the upregulation of TCF3, ETV5, FOXM1, CAMTA1, CEBPB, CBFB, and NFE2L3; SOX4 is related to the overregulation of TCF3, SOX9, CEBPB, NFE2L3, HMGB3, JUN, and HES6; and FOXM1 is related to the overregulation of TCF3, TEAD4, SOX4, ETV4, MYC, ELK1, ARNTL2, HMGA1, and PHF19. The co-regulatory analysis suggests the formation of protein integration complexes between overregulated TFs (CAMTA1, CBFB, CEBPB, ETV4, FOXM1, HES6, JUN, MYC, NFE2L3, PHF19, SOX4, SOX9, and TEAD4) controls transcriptional regulatory function during the acquisition of the hallmarks of cancer.

#### 3.2.3 Gastric cancer

**Figure 5:**
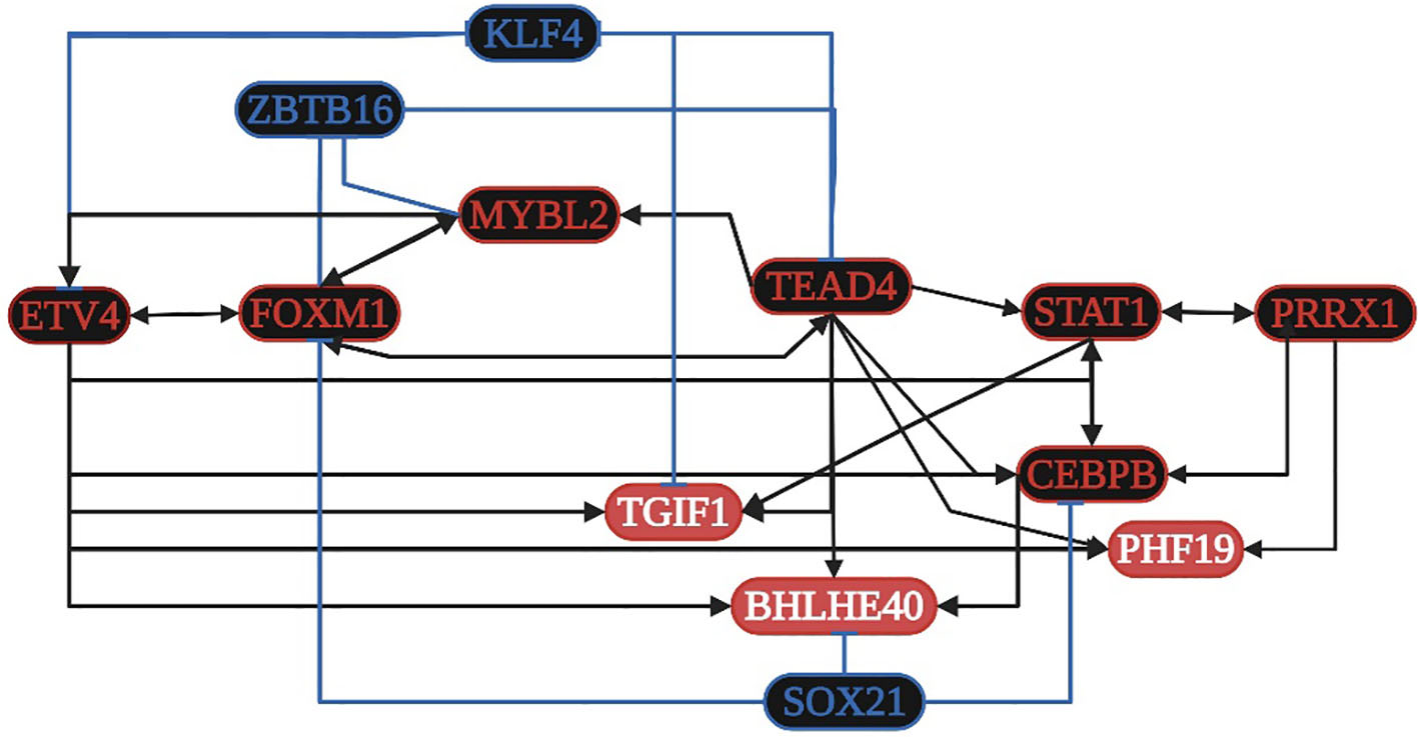
Transcriptional regulatory network (TRN) of common transcription factors (TFs) in gastric cancer. Downregulated TFs are in blue, upregulated TFs are in red, and the key overregulated and downregulated TFs are in black. The black pointed arrows represent overregulation, and the blue flat arrows represent downregulation. Created with BioRender.com.

STAT1 is overregulated in seven datasets and is related to: 1) angiogenesis; 2) the formation of the macromolecular complex; 3) type I interferon, cytokine-mediated signaling, and pathways in cancer; and 4) Kaposi sarcoma-associated herpes (KSH) and HPV infection. CEBPB is overregulated in seven datasets and is related to 1) the regulation of the inflammatory response, 2) the cellular response to amino acid stimulus, and 3) the TNF and IL-17 signaling pathways. TGIF1 is overregulated in seven datasets and is related to 1) the cellular response to growth factor stimulus and 2) the TGF-beta signaling pathway. FOXM1 is overregulated in seven datasets and is related to 1) the regulation of cell proliferation and 2) the cellular senescence along with MYBL2. TEAD4, ETV4, and PRRX1 are overexpressed in nine and seven of the ten datasets analyzed, but they are not in DAVID´s annotation analysis (**Supplementary Table 3**). According to the TRN analysis (**Figure 5**), TEAD4 and ETV4 are related to the overregulation of STAT1, MYBL2, CEBPB, PHF19, BHLHE40, TGIF1, and FOXM1. PRRX1 is related to the overregulation of STAT1 and CEBPB. FOXM1 is related to the overregulation of TEAD4 and MYBL2. STAT1 is related to the overregulation of PRRX1 and CEBPB. MYBL2 is related to the overregulation of ETV4 and FOXM1. CEBPB is related to the overregulation of STAT1 and BHLHE40. TEAD4 is also related to the downregulation of KLF4, whereas FOXM1 and MYBL2 are related to the downregulation of ZBTB16. The co-regulatory analysis suggests that the formation of protein-protein interaction complexes between eight overregulated TFs (BHLHE40, ETV4, FOXM1, MYBL2, PHF19, PRRX1, STAT1, and TEAD4) controls transcriptional regulatory function.

## 4 Discussion

Bioinformatic analysis of high-throughput sequencing methodologies, such as microarrays and RNA sequencing, allows the identification of all the transcriptionally deregulated genes (DEGs) involved in the modulation of biological processes and signaling pathways related to tumor cells grade, differentiation status, metastatic potential, and patients’ survival (Otálora-Otálora et al., 2019, Otálora-Otálora et al., 2023a). The human genome has information for approximately 1,400 regulatory genes known as TFs, representing approximately 6% of all human protein coding genes. DNA-binding TFs recognize cis-regulatory elements of target genes, which make them the most direct regulators of gene transcription during cellular differentiation, development, and the response to external factors through the activation and/or inhibition of specific signaling pathways (Weidemüller et al., 2021). In normal cells and tumor cells, TF coding genes can be regulated positively or negatively by genetic and epigenetic mechanisms (**Figure 1**) that control protein localization on the binding site, resulting in a loss or gain of function (Whiteside and Goodbourn, 1993; Filtz et al., 2014).

**Figure 6:**
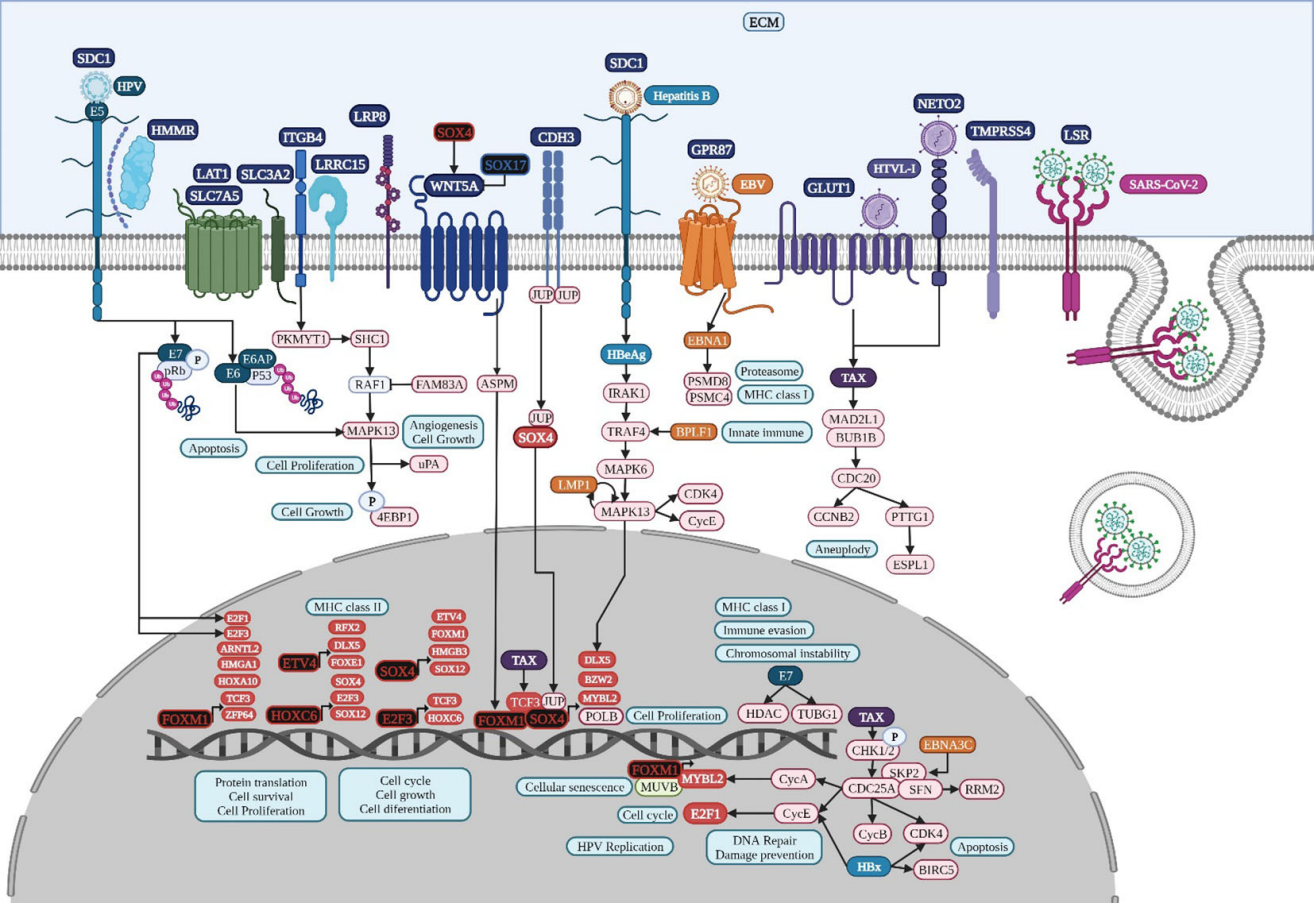
Microbiome interaction with the membrane receptors of lung cancer-related cells, activating signaling pathways involved in transcriptional regulation during lung tumorigenesis. Upregulated genes and TFs are in red and the key upregulated TFs are in black. Created with BioRender.com.

The global transcriptomic network analysis highlighted the impact of five TFs, SOX4, TCF3, TEAD4, ETV4, and FOXM1, in gut and lung cancer (**Figure 2**). SOX4 is an important developmental TF known to regulate stemness, differentiation, progenitor development, and signaling pathways, including the TGF-β, p53, PI3K-Akt, and Wnt pathways, which strengthen its expression (Moreno, 2020). SOX4 shows increased expression in NSCLC tissues, which is specifically correlated with differentiated degree status, the clinical stage, T classification, N classification, M classification, and poor overall patient survival (Wang et al., 2015). *SOX4* is a known target gene of the TGF-β signaling pathway, via the direct binding of SMAD2/3 in complex with SMAD4 to the *SOX4* gene promoter region, and has been shown to be related to the regulation of neural-related nature target genes in SCLC lung tumors with neuroendocrine characteristics (Castillo et al., 2012). In lung cancer, *BMP5* is a common gene but it is downregulated, as are TGF-β receptors and SMADs; therefore, none of them may be involved in the regulation of SOX4 and DLX5 expression. However, according to the TRN analysis, SOX4 can be regulated by HOXC6, whereas DLX5 can be regulated by SOX4, FOXM1, and ETV4 (**Figure 3**); therefore, its regulation might be controlled by the pathways related to these TFs. In colon cancer, *BMP7* is common overregulated gene, as are *TGFB2*, *TGFBI*, and *TGIF2*, which might be involved in the regulation of SOX4 (**Supplementary Table 2**). Junction plakoglobin (*JUP*), also known as γ-catenin, a major component of the submembrane of adherens junctions and desmosomes in mammalian cells, can interact with cadherin 3 in adherens junctions in the cytoplasmic component; both are common overregulated genes in lung cancer (**Supplementary Table 1**) and bind to SOX4 via two trypsinized fragments (**Figure 6**), while Wnt signaling induces the nuclear colocalization of SOX4 and JUP (Lai et al., 2011). In lung cancer, SOX4 expression is probably related to the overregulation of WNT5A; then, SOX4, FOXM1, TCF3, and JUP might form a stable protein complex with other factors and co-factors for the regulation of transcriptional targets (**Figure 6**). SOX4 overexpression promotes sphere formation and the self-renewal of colorectal cancer cells (**Figure 2**), which directly bind to the HDAC1 promoter, encouraging HDAC1 transcription and thereby stem cell maintenance, Wnt, Notch, the cell cycle, and transcriptional misregulation pathways in cancer (Liu et al., 2021b). Forkhead box protein M1 (FOXM1) is overexpressed in NSCLC and SCLC, is related to the regulation tumorigenesis, cell cycle progression,cancer therapy resistance, and metastasis, as it can translocate to the nucleus and bind to the regulatory regions of several target genes crucial for the survival of cancer cells (Zhang et al., 2015; Liang et al., 2021). FOXM1 is a key cell cycle regulator that plays a key role in embryogenesis and cell proliferation and has been strongly linked to solid tumors like colon cancer, where it is linked to reduced disease-free survival, which suggests it is an important prognostic marker (Rather et al., 2023). Furthermore, in gastric cancer, FOXM1 is related to proliferation and invasion, and when it is co-expressed with hTERT it might be involved in cell-cycle-related pathways and positively related to advanced stages and poor outcomes (Tang et al., 2023). In the transcriptomic analysis, there were three other common DEGs (*NEK2*, *GREM1*, and *HSP90AB1*) related to polymerase activity (**Supplementary Table 3**) that might be related to the process.

The transcription factor 3 gene (*TCF3*) is a key TF overexpressed in lung cancer (**Figure 2**) and is mainly involved in the cell cycle, cell division, and proliferation (Zhang et al., 2018). TCF3 is primarily a transcriptional repressor (Gregorieff and Clevers, 2005) and is not expressed in adult intestine (Wong et al., 2002). Non-canonical WNT signaling could be related to TCF3 upregulation in colon cancer (**Figure 4**), along with other key overregulated TFs, and the formation of transcriptional regulatory complexes according to DAVID´s analysis (**Supplementary Table 2**); therefore, it might be involved in the control of gene expression for the acquisition of the hallmarks of cancer (**Figure 1**). TCF3 (E2A immunoglobulin enhancer-binding factors E12/E47) E12 forms heterodimers with other basic helix-loop-helix proteins during cell differentiation, whereas E47 can homo and heterodimerize, promoting tumor angiogenesis and proliferation (Peinado et al., 2004); both act as transcriptional repressors of E-cadherin during EMT, which is linked to tumor aggressiveness (Perez-Moreno et al., 2001). TCF3 upregulation is caused by promoter hypomethylation during development, colon cancer progression (Li et al., 2014), and the transcriptional upregulation of multiple cyclin-dependent kinase inhibitors like CDKN1A, p15INK4B, and p16INK4B (Pagliuca et al., 2000), and probably CDKN3 in colon cancer (**Supplementary Table 2**). According to the RTN analysis, the overregulation of TCF3 might be controlled by TEAD4, SOX4, CEBPB, and FOXM1 (**Figure 4**).

**Figure 7:**
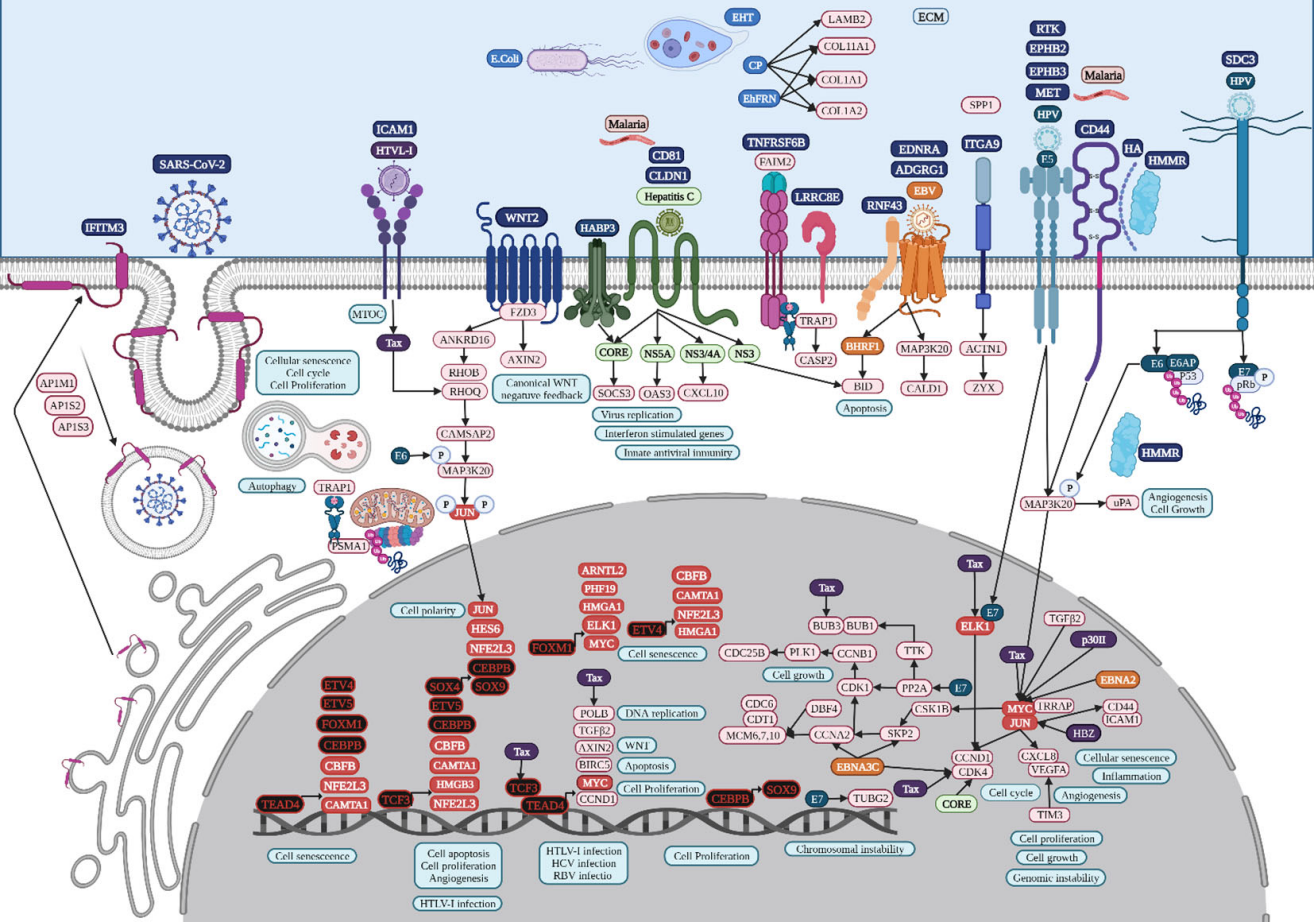
Microbiome interaction with the membrane receptors of colon cancer-related cells, activating signaling pathways involved in transcriptional regulation during colon tumorigenesis. Upregulated genes and TFs are in red and the key upregulated TFs are in black. Created with BioRender.com.

TEAD4 belongs to a transcriptional enhancer activator domain family of TFs, has a profound impact on physiological and pathological processes through gene expression regulation during cell survival, cell proliferation, tissue regeneration, and stem cell maintenance (Gu et al., 2020), controls chemoresistance, promotes EMT, ECM remodeling, the secretion of paracrine factors, and heterotypic cellular communication, and is related to several signaling pathways (Liu et al., 2024). According to the RTN analysis, the overregulation of TEAD4 might be controlled by TCF3 and FOXM1 (**Figure 4**). In colon cancer, TEAD4 may also form a complex with TCF3 under the regulation of Wnt (Jiao et al., 2017). The non-canonical Wnt signaling pathway might be involved in colon cancer progression (**Figure 7**), specifically in planar cell polarity (PCP), in which WNT2 binds to FZD3, a frizzled transmembrane receptor, to activate ankyrin repeat domain 16 (ANKRD16), ras homolog gene family members B and Q (*RHOB* and *RHOQ*), and a mitogen-activated protein kinase, MAP3K20, modifying JUN phosphorylation and its transcriptional regulatory function, which affects cell polarity and cytoskeleton organization (Gómez-Orte et al., 2013). In colon cancer, WNT2 might bind to FZD3 to upregulate AXIN2, as well as RNF43 for its negative feedback regulation (Jho et al., 2002), in which the modification and degradation of β-catenin are key pathway events in tumor progression (Zhao et al., 2022).

**Figure 8:**
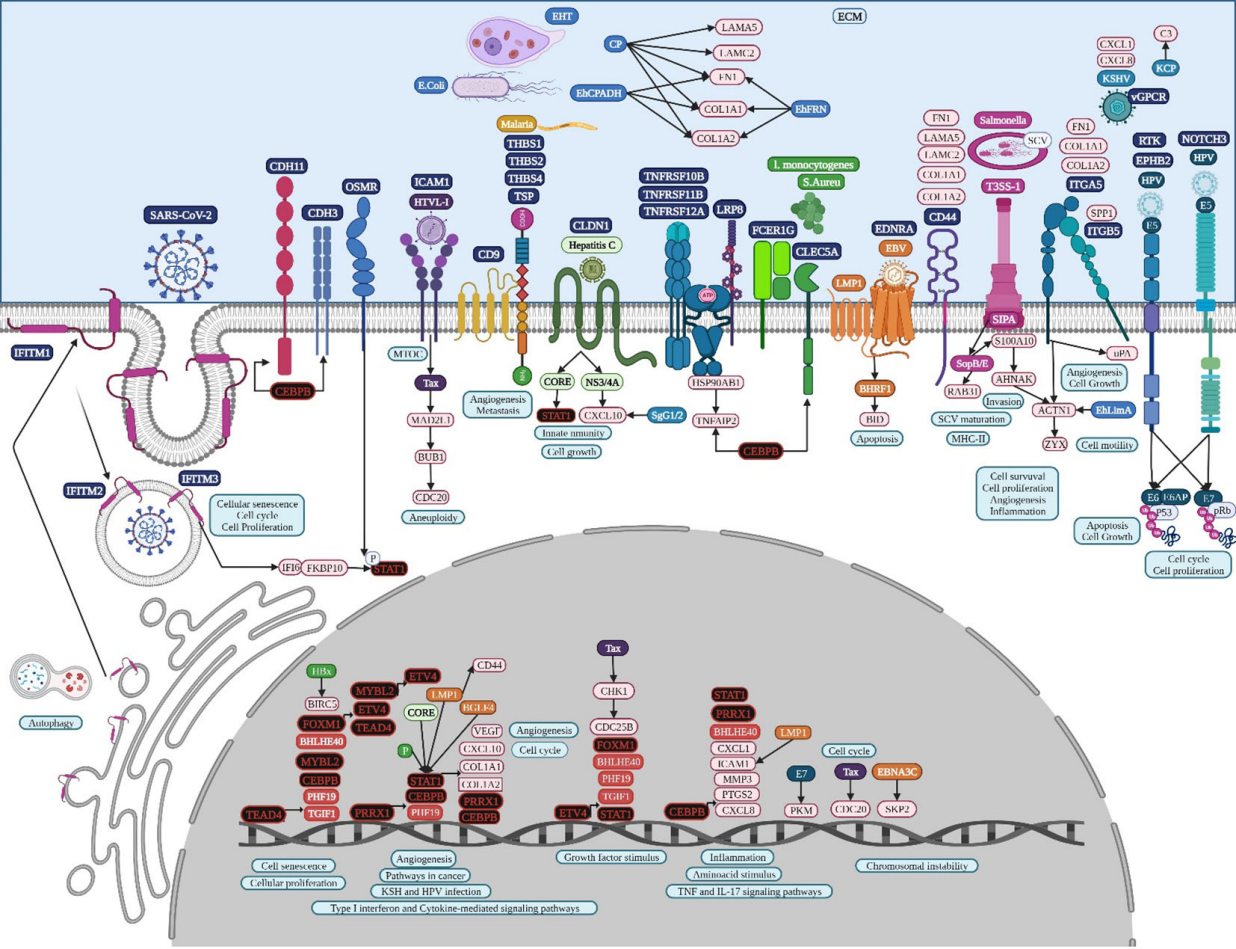
Microbiome interaction with the membrane receptors of gastric cancer-related cells, activating signaling pathways involved in transcriptional regulation during gastric tumorigenesis. Upregulated genes and TFs are in red and the key upregulated TFs are in black. Created with BioRender.com.

TEAD4 stimulates the glycolysis and proliferation of gastric cancer cells (Zhan et al., 2023). The TNF-α-ERK-VGLL1-TEAD4 pathway upregulates integrin αV expression, increasing the adhesion and invasive ability of gastric cancer cells (Hwang et al., 2022). ETV4 is a TF of the E26 transformation‐specific (ETS) family and plays an important role in tissue development, promoting the growth and metastasis of gastric cancer (Lu et al., 2019). ETV4 is also significantly related to growth at the advanced stage, lymph node metastasis, and the poor prognosis of NSCLCs, with a direct regulatory effect on matrix metalloproteinase 1 (*MMP1*), which is a common overregulated gene (**Supplementary Table 1**) related to cell proliferation and migration, and its co-overexpression is associated with a poor prognosis in human NSCLCs, suggesting that it could be a useful biomarker of tumor progression and worse outcomes in NSCLCs (Wang et al., 2020). Moreover, ETV4 is one of the most expressed genes in colon adenocarcinoma, and it is related to cell proliferation, colony formation, and cell migration (Fonseca et al., 2021). ETV4 is a transcription activator of TNF‐α, promoting hepatic inflammation in hepatocellular carcinoma (Qi et al., 2023). Several hallmark pathways are significantly enriched in patients with high PRRX1, including IFN-γ response, IL6-Jak-Stat3 signaling, TNF-α signaling via NF-kB, and IFN-α response in uveal melanoma cancer patients (Meng et al., 2022b). There is evidence that suggests an important role of TEAD4, ETV4, and PRRX1 regulatory activity, the main TFs identified in the TRN analysis (**Figure 2**), during the TNF and IFN singling pathway in gastric tumorigenesis (**Figure 8**), along with STAT1 and CEBPB, that might be validated experimentally, to establish their importance during the acquisition of the hallmarks of cancer. Signal transducer and activator of transcription 1 (STAT1) has been linked to anti-tumor immune responses, and *H. pylori* might be involved in the regulation of its expression (Li et al., 2022). In gastric cancer, elevated alpha-inducible protein 6 (IFI6) and FKBP prolyl isomerase 10 (FKBP10) (**Supplementary Table 3**) could be responsible for the activation of STAT1 expression, diminishing antiviral responses (James et al., 2020).

Transcriptomic studies of gut and lung cancer have been performed and analyzed by several scientific groups around the world. In colon cancer, there are transcriptomic analyses comparing tumor cases and healthy controls, identifying the differences in global transcriptional regulatory programs, showing a large reduction of transcriptional interactions in tumor networks, TFs, and target genes, while the average gene expression is conserved and some TFs increased their connectivity (Cordero et al., 2014). One study analyzed the signatures of co-deregulated genes and their transcriptional regulators in five datasets, highlighting 17 hub co-upregulated genes and 18 hub co-downregulated genes, including three well-known TFs and three kinases, as critical genes in colon cancer (Mastrogamvraki and Zaravinos, 2020). In gastric cancer, there are transcriptomic analyses comparing tumor cases and healthy controls, in which microarray datasets were analyzed to make a protein-protein interaction network with the retrieval of an interacting genes database and TCGA-STAD datasets, identifying the top TFs according to the calculation of the regulatory impact factor (Wang, 2017) (Zhao et al., 2021). An algorithm named mRBioM was even developed for the identification of potential mRNA biomarkers from the complete transcriptomic RNA profiles of gastric adenocarcinoma (Dong et al., 2021), of which some were TFs and cell receptors; however, it did not identify the main regulators of transcription related to the acquisition of the hallmarks of cancer or suggest any other biological processes in which all might be involved during the tumorigenic process. In our lung cancer bioinformatic analysis of TF RUNX2 (Otálora-Otálora et al., 2022a), we analyzed and compared our pipeline and results (Otálora-Otálora et al., 2019, Otálora-Otálora et al., 2022b) with previous studies, finding eleven publications assessing the lung cancer TRN with different microarray and RNA-Seq studies, and performing direct and very different bioinformatics analyses on datasets created or selected from the databases. None attempted to perform a global analysis of lung cancer, and most conducted the analysis with a reduced number of datasets for each subtype of lung cancer independently. None of them tried to select deregulated genes unique to lung cancer that are not deregulated in other lung diseases or other types of cancer, and none performed a joint co-regulatory analysis to study the cooperative and coordinated regulatory functions of TFs. However, TFs identified using our bioinformatic pipelines are also in some of the other studies but their regulatory functions during tumor processes were not deeply analyzed. Therefore, regardless of the cell types, the detection methodology of gene expression, and the bioinformatics methodology used, there is a group of regulatory genes or TFs (Otálora-Otálora et al., 2023a).

DAVID´s gene enrichment analyses in lung, colon, and gastric cancer highlights the activation of tumorigenic signaling pathways through the interaction of specific cancer-cell membrane receptors with several microorganisms, including HTLV-1, HPV, EBV, and SARS−CoV−2, which might be related to the establishment of the TRN of TFs in lung cancer (**Figure 3**), colon cancer (**Figure 4**), and gastric cancer (**Figure 5**), and these may be related to the formation of the microbiome network, controlling the gene expression regulation of the specific cancer-cell membrane receptors (**Table 2**). HTLV-1 is an enveloped single stranded RNA human deltaretrovirus that causes lifelong infection of CD4+ and CD8+ T-cells, monocytes, and other lymphoid and non-lymphoid cells via the ubiquitous glucose transporter-1 (GLUT1) and neuropilin (Einsiedel et al., 2021). *GLUT1*, also known as solute carrier family 2 (*SLC2A1*), is a common overregulated gene in lung cancer that might also function as a receptor for HTLV-1 (Manel et al., 2003). GLUT1 is a transmembrane protein involved in passive glucose transport and has a high glucose affinity compared with other transporters; therefore, it might be key in tissues where glucose is the main energy source (Kokeza et al., 2023). GLUT1 is overexpressed in various solid and hematological malignancies, such as colorectal carcinomas, gastrointestinal stromal tumors, and lung cancers (Zhang et al., 2019), but the correlation with the grade and stage of the tumor and the clinical outcome has not been defined yet (Schuurbiers et al., 2014). The HTLV-1 transactivator protein Tax is a potent activator of a variety of transcription pathways and interacts with cell cycle components dysregulating normal cell cycle controls, leading to several cellular abnormalities, including aneuploidy, the regulation of TCF3 (**Figure 6**), and the immortalization of T-cells, all of which play key roles in oncogenesis (Currer et al., 2012). Increased CHK1/2 activity by Tax during S phase restricts the CDK-dependent phosphorylation of FOXM1, preventing the premature expression of G2/M genes, including those encoding cyclin-dependent kinase 4, cyclin B1, and cyclin A2 (Branigan et al., 2021), all common DEGs in lung cancer (**Supplementary Table 1**). In lung cancer, cyclin-dependent kinase 3 may inactivate retinoblastoma and DREAM through phosphorylation for the temporal control of expression of two gene sets: the expression of the first gene set peaks in G1/S for DNA synthesis, and it might be activated by E2F1; the second set reaches maximum expression during G2/M for mitosis, coordinated by MuvB, MYBL2, and FOXM1 (Fischer et al., 2022), a complex that regulates cell cycle progression and entry into senescence (**Figure 6**). *NETO2* (Neuropilin and tolloid-like 2) is a common overexpressed gene in lung cancer that encodes a transmembrane protein containing two extracellular CUB domains followed by a low-density lipoprotein class A domain (**Figure 6**), which may also be related to HTLV-1 infection, as it has been associated with clinical stage and lymph node metastasis, cell proliferation, apoptosis, tumor growth, migration, and EMT, increasing the phosphorylation of ERK (Xu et al., 2021a).

**Table 2:** Cancer-cell membrane receptors related to the interaction of key microorganisms according to KEGG signaling pathways and DAVID´s annotation analysis, and the regulation of their gene expression by key transcription factors.

cancer-cell membrane receptor	Expression regulated by Transcription factorS	Microorganism	Type of cancer
**Glucose transporter-1 (GLUT1)** **solute carrier family 2 (SLC2A1)**	SOX4, BZW2, FOXM1, HOXC6, HMGA1, and HMGB3	HTLV-1	Lung
**Neuropilin and tolloid-like 2 (NETO2)**	DLX5 and HMGB3	HTLV-1	Lung
**Intercellular adhesion molecule-1 (ICAM-1)**	TEAD4, TCF3, ETV4, ETV5, FOXM1, SOX4, and SOX9	HTLV-1	Colon
	TEAD4, PHF19, PRRX1, BHLHE40, MYBL2, FOXM1, ETV4, and STAT1	HTLV-1	Gastric
**Syndecan 1 (SDC1)**	DLX5, ETV4, FOXM1, and HMGB3	HPV	Lung
**Syndecan 3 (SDC3)**	TEAD4, TCF3, ETV4, ETV5, FOXM1, SOX4, and SOX9	HPV	Colon
**MET proto-oncogene, receptor tyrosine kinase (MET)**	TEAD4, TCF3, ETV4, ETV5, FOXM1, SOX4, and SOX9	HPV	Colon
**EPH receptor B2 (EPHB2)**	PRRX1, TEAD4, ETV4, and FOXM1	HPV	Gastric
**G Protein-Coupled Receptor 87 (GPR87)**	FOXM1, HOXC6, HMGB3, and E2F3	EBV	Lung
**Endothelin Receptor Type A (EDNRA)**	TEAD4, TCF3, ETV4, ETV5, FOXM1, SOX4, and SOX9	EBV	Colon
	PRRX1, BHLHE40, ETV4, and STAT1	EBV	Gastric
**Adhesion G protein-coupled receptor G1 (ADGRG1)**	TEAD4, TCF3, ETV4, ETV5, FOXM1, SOX4, and SOX9	EBV	Colon
**Lipolysis-stimulated lipoprotein receptor (LSR)**	ETV4, FOXM1, HOXC6, and HMGB3	CD	Lung
		SARS−CoV−2	Lung
**Interferon-induced transmembrane protein 3 (IFITM3)**	TEAD4, TCF3, ETV4, ETV5, FOXM1, SOX4, and SOX9	SARS−CoV−2	Colon
**Interferon-induced transmembrane protein 1,2,3 (IFITM1,2,3)**	TEAD4, ETV4, PRRX1, and FOXM1	SARS−CoV−2	Gastric
**Integrin beta 4 (ITGB4)**	FOXM1, HOXC6, HMGB3, and HMGA1	HCV	Lung
**Claudin-1 (CLDN1)**	TEAD4, TCF3, ETV4, ETV5, FOXM1, SOX4, and SOX9	HCV	Colon
**Integrin-α9 (ITGA9)**	TEAD4, TCF3, ETV4, ETV5, FOXM1, SOX4, and SOX9	EHT	Colon
**Integrin subunit alpha 5 (ITGA5)**	PRRX1, PHF19, TEAD4, FOXM1, and ETV4	EHT	Gastric
**Integrin subunit beta 5 (ITGB5)**	PRRX1, PHF19, TEAD4, FOXM1, and MYBL2	EHT	Gastric
**Integrin subunit beta like 1 (ITGBL1)**	PRRX1	EHT	Gastric
**Hyaluronan-mediated motility receptor (HMMR)**	SOX4, FOXM1, HOXC6, HMGB3, E2F3, HOXA10, E2F1, and DLX5	HBV	Lung
**Large amino acid transporter 1 (LAT1 or SLC7A5)**	SOX4, DLX5, BZW2, FOXM1, HMGB3, and HMGA1	HBV	Lung
**S100 calcium binding protein A10 (S100A10)**	PRRX1, PHF19, TEAD4, FOXM1, and MYBL2	SEST	Gastric

HTLV-1 spreads directly between lymphocytes and other cells via a specialized cell-cell contact, named the virological synapse, which is accompanied by the orientation of the microtubule-organizing center in the infected T cell toward the cell contact region with the non-infected target cell, followed by intracellular Tax protein expression and the stimulation of intercellular adhesion molecule-1 (ICAM-1) on the cell surface to trigger microtubule organization center polarization in the HTLV-1–infected colon cell (**Figure 7**), key in the migration and activation of signaling pathways (Nejmeddine et al., 2009). HTLV-1 Tax acts on genes indirectly by binding several TFs, such as TCF3, ELK1, and MYC in colon cancer, as well as POLB, BUB3, and CDK4, according to the HTLV-1 infection signaling pathway (**Figure 7**). HTLV-1 HBZ might bind to JunD, possibly to mediate the activation of ICAM-1 expression, which increases the efficiency of HTLV-1 infection (Fazio et al., 2019). HTLV-1 pX ORF II encodes two proteins, p13II and p30II, multifunctional regulators that modulate Tax-responsive element-mediated transcription and repress cell and viral gene expression to favor cell survival (Green, 2004), which deregulates host signaling pathways involved in aberrant cell growth and proliferation through the induction of lysine-acetylation of c-MYC oncoprotein (**Figure 7**) and the inhibition of apoptosis, contributing to HTLV-1-induced carcinogenesis (Romeo et al., 2015).

HTLV-1 may spread between gastric cancer cells, followed by the combination of intracellular Tax protein expression and the stimulation of ICAM-1 on the cell surface to trigger microtubule organization center polarization (Nejmeddine et al., 2009) (**Figure 8**). HTLV-1 Tax directly targets G2 and mitotic regulator hsMAD1, activates mitotic spindle checkpoint function following chromosomal mis-segregation, and controls the G1/S check point, resulting in aberrant anaphase progression, chromosomal instability, DNA aneuploidy, and continuous cellular proliferation (de la Fuente et al., 2006). Tax promotes activation of the anaphase promoting complex (APC)-APCCdc20p, leading to a reduction in Pds1p/securin and Clb2p/cyclin B levels (Liu et al., 2003). Tax represses cellular DNA repair by binding to Chk2 and Chk1, impairing kinase activities *in vitro* and *in vivo*, silencing cellular checkpoints, which guard against DNA structural damage and chromosomal mis-segregation, thus increasing the appearance of a mutant phenotype and perturbing dynamic complexes that coordinate the processes of cell cycle regulation and DNA repair (Park et al., 2004).

HPV is a family of more than 200 small non-enveloped double-stranded circular DNA viruses that can be subdivided in two main groups: high risk and low risk, based on their ability to induce several types of cancers, including lung carcinomas in smoker and non-smoker subjects (Osorio et al., 2022b). HPV infects the basal cells of the mucosal epithelium through capsid proteins L1 and L2, which induce internalization of the virus, probably through the attachment to syndecan 1 (SDC1) (Ozbun and Campos, 2021). Early genomic regions E1 and E2 are the first transcribed for HPV genome amplification; E2 protein is a TF, containing viral DNA binding and transactivation domains (McBride, 2013), E5 is a multipass protein that activates receptors and induces proliferation, E6 inhibits apoptosis by interacting with p53, inducing its degradation and increasing telomerase activity, and E7 binds to protein phosphatase 2A subunits, releasing and activating TFs like E2F involved in cell cycle progression (**Figure 6**). Then, E6 and E7 induce genome amplification and uncontrolled proliferation in growth arrested differentiated cells, increasing the infected area to finally package HPV genome into L1 and L2 capsid protein and exit cells, which have lost nuclear and cytoplasmic integrity, aided by the E4 protein, which disrupts cytokeratin filaments (Medda et al., 2021). E7 oncoprotein rapidly induces centrosome abnormalities thereby causing the formation of supernumerary mitotic spindle poles and increasing the risk of chromosome mis-segregation (**Figure 6**) through a process that involves an increase in PLK4 mRNA steady-state levels (Korzeniewski et al., 2011) and tubulin gamma 1 (Starita et al., 2004), which are both overregulated common genes in lung cancer (**Supplementary Table 1**). HPV16 E7 recruits histone deacetylase HDAC1 and histone demethylase JARID1B or KDM5B to the regulatory region upstream of the TLR9 transcriptional start site and reduces H4 acetylation and H3K4me3, leading to the downregulation of TLR9 expression and the evasion of innate immune responses (Hasan et al., 2013). The treatment of HPV-positive tumor cells with an HDAC inhibitor increases the surface expression of the major histocompatibility complex class I (MHC-I) molecules, increasing the susceptibility of tumor cells to E7-specific CD8+ T cells (Lee et al., 2013). HPV oncoprotein E7 interacts with the DNA methyltransferase DNMT1, an overregulated common gene in lung cancer (**Supplementary Table 1**), stimulating its methyltransferase activity (Burgers et al., 2007) to induce epigenetic reprogramming in tumor cells.

HPV capsid proteins L1 and L2 may induce virus internalization, probably through the attachment to syndecan 3 (*SDC3*) (**Figure 7**), a known HPV-binding receptor during colon tumorigenic processes (Mukherjee et al., 2023) and a common overregulated gene in colon cancer. *SDC3* could also be a WNT2 receptor in colon cancer and a potential regulator for the development of chronic inflammation (Chen et al., 2022). Early HPV genomic regions E1, E2, E5, E6, and E7 might be fulfilling some of the same functions as lung cancer (Medda et al., 2021). Furthermore, E6 can selectively upregulate WNT4, JIP1, and JIP2 translation, resulting in the activation of the non-canonical WNT-PCP-JNK pathway through the phosphorylation of mitogen-activated protein kinase (**Figure 7**) to promote cell proliferation and tumor growth (Zhao et al., 2019). HPV may also bypass IFITM restriction and use certain IFN-inducible proteins to facilitate virus infection (Westrich et al., 2017). HPV16 E5 may increase MET levels, a growth factor receptor critical for tumor cell invasion, motility, and cancer metastasis (Scott et al., 2018). Furthermore, MET induction by E5 requires EGFR, which is also increased by E5 at the mRNA level. Crosstalk between c-MET and various membrane protein partners, including the EGFR, α6β4 integrin, and CD44, results in additional signaling response modulation (Raj et al., 2022). CD44 upregulation enables cell self-renewal, inflammation, and migration at multiple stages and is related to a poor prognosis (Wang et al., 2019). Cell surface interaction between HMMR, CD44, HA, and tyrosine kinases activates MAP kinase cascade, which, in absence of intracellular HMMR, can regulate a mitogenic response involved in cell proliferation and random motility, and in the presence of intracellular HMMR, MAPKs bind to protein partners, which allows HMMR to enter the nucleus to regulate the expression of MYC, controlling its stabilization via AURKA (Otto et al., 2009), microtubule dynamics via the centrosome, and cell cycle progression also via AURKA, a targeting protein for TPX2, hence controlling the expression of genes involved in cell motility, such as matrix metallopeptidases (Raj et al., 2022). HPV capsid proteins L1 and L2 may induce virus internalization, probably through the attachment to neurogenic locus notch homolog protein 3 (NOTCH3) or EPH receptor B2 (EPHB2) in gastric cancer (**Figure 8**). HPV E7 may regulate the formation of TF complexes to control cell cycle progression and promote aberrant cellular proliferation (Das et al., 2021), as suggested by the intersect of HPV and the NOTCH signaling pathway in gastric cancer (Xie and Yan, 2023). NOTCH3 is associated with more aggressive disease and poor prognosis, acting as a molecular switch in angiogenesis and the release from tumor dormancy (Inder et al., 2017). In cancer cells, EPHB2 may promote EMT (Gao et al., 2014), and in small extracellular vesicles, EPHB2 may promote angiogenesis, inducing ephrin-B reverse signaling, and STAT phosphorylation (Sato et al., 2019).

EBV is a ubiquitous gamma herpesvirus that causes persistent infections and some lymphoid and epithelial tumors (Osorio et al., 2022a) and might use G protein-coupled receptor (GPCR) signaling (Zhang et al., 2016), like GPR87, a common overregulated DEG in lung cancer (**Figure 6**). Viral GPCRs are composed of seven membrane-spanning helices and intracellular and extracellular domains and have a ligand-independent signaling capacity or constitutive activation, but behave like human chemokine receptors, but behave like human chemokine receptors to guide immune cells to the site of inflammation and participate in tumor cells survival, growth, and metastasis (Zhang et al., 2016). The EBV open reading frame (BILF1) evades the host immune system by downregulating MHC class I and is capable of inducing signaling-mediated tumorigenesis (Fares et al., 2019). The N-terminal part of the large BPLF1 protein contains the catalytic site for ubiquitin ligase and deubiquitinase activity and suppresses TLR-mediated activation as a mechanism to counteract the innate antiviral immunity of infected hosts (van Gent et al., 2014). EBV nuclear antigen 1 (EBNA1) is required for the replication and maintenance of the EBV’s extrachromosomal genome in the host cell, with a GAr signal (glycine and alanine rich segment) that interferes with a protein’s degradation and allows the virus to escape host immunity via the MHC class I pathway (Finley, 2009). The EBV-encoded *LMP1* oncogene is involved in the transformation, proliferation, and metastasis of several EBV-associated tumors, which are related to its ability to upregulate anti-apoptotic proteins and growth signals and the expression of p38 (MAPK13) in response to stimuli such as stress or primary infection that lead to an increase in LMP1 promoter activity, and may allow the cells to escape apoptosis, suggesting the presence of a positive autoregulatory loop in LMP1 upregulation (Johansson et al., 2010). EBV nuclear antigen (EBNA)3C functions as a transcriptional regulator by interacting with several well-known cellular and viral TFs (Kumar et al., 2009). EBNA3C recruits SKP2 E3 ligase activity to facilitate the degradation of p27KIP1 and pRb, and interacts with cyclin A, cyclin D1, p53, E2F1, and CHK2 (Bhattacharjee et al., 2016). EBV might use G protein-coupled receptor (GPCR) signaling (Zhang et al., 2016), like EDNRA and ADGRG1, which are common DEGs in colon cancer. EBV Bam HI fragment H rightward open reading frame (BHRF1) is a viral homolog of cellular BCL-2 pro-survival proteins (vBCL-2s) and confers strong resistance to diverse apoptotic stimuli and interacts with the cellular pro-apoptotic BCL-2 protein BID in colon cancer (**Figure 7**) to inhibit DNA-damage-induced apoptosis (Fitzsimmons et al., 2020). EBV EBNA2 induces transcription of the MYC oncogene and decreases lytic EBV replication (Münz, 2019). EBNA3C stabilizes c-Myc and recruits c-Myc and its co-factor Skp2 to c-Myc-dependent promoters, which result in increased c-Myc-dependent transcription (Kumar et al., 2009). Cyclin A, an activator of S phase progression, binds tightly to EBNA3C to stimulate cyclin A-dependent kinase activity and cell cycle progression (Knight and Robertson, 2004). EBNA3C increases the activity of the cyclin D1-CDK4 complex toward histone H1 and a truncated mutant of pRb, increasing pRb poly-ubiquitination and thereby increasing its degradation and abolishing its growth suppressive function (Saha et al., 2011).

EBV also uses GPCR signaling (Zhang et al., 2016), such as *EDNRA*, a common DEG in gastric cancer. BHRF1 also interacts with the cellular pro-apoptotic BCL-2 protein BID (**Figure 8**) to inhibit DNA-damage-induced apoptosis (Fitzsimmons et al., 2020). EBNA3C may also function in gastric cancer as a transcriptional regulator by interacting with several well-known cellular and viral TFs (Kumar et al., 2009), and recruits SKP2 to facilitate the degradation of p27KIP1 and pRb (Bhattacharjee et al., 2016). EBV LMP1 expression in gastric carcinomas may lead to tumor growth avoiding its apoptotic effects and immunologically mediated elimination (Sheu et al., 1998). LMP1 induces STAT1 expression, which probably induces tyrosine phosphorylation depending on the secretion of IFNs (Najjar et al., 2005), and transcriptional activity mediated by the specialized C-terminal activating region 1 or 2 cytoplasmic domains of LMP-1 (Richardson et al., 2003). LMP1 induces the expression of CD44 on the cell surface, a molecule implicated in increased tumor growth and dissemination (Zhu et al., 2021). EBV BGLF4 protein kinase may have a similar function as cellular cyclin-dependent kinase, regulating multiple cellular and viral substrates, which represses the poly(I:C)-stimulated expression of endogenous IFN-beta mRNA and the phosphorylation of STAT1 at Tyr701, which promotes the expression of downstream genes, suppressing host innate immune responses and facilitating virus replication (Wang et al., 2009). The C-terminal activator region 1 of LMP1 delivers a cooperating signal to induce ICAM1 mRNA in response to various inflammatory mediators, including bacterial lipopolysaccharide, phorbol esters, oxidant stress, and pro-inflammatory cytokines, such as TNFα, IL-1β, and γ-IFN (Mehl et al., 2001).

The lipolysis-stimulated lipoprotein receptor (LSR) is the target molecule for cell binding and the internalization of *Clostridium difficile* and might cooperate with the LDL receptors (Yen et al., 2008) to regulate cell proliferation, invasion, and migration (Zhang and Ma, 2021), probably via MAPK signaling (Nagase et al., 2022). LSR may also activate the SARS−CoV−2 S proteins and increase the viral infection of lung cancer cells (Gong et al., 2023), along with host co-receptors that might be involved in cell infection, such as NETO2, GRP87, and transmembrane protease serine 4 (TMPRSS4) (Avdonin et al., 2023) (**Figure 6**). Interferon-induced transmembrane protein 3 (*IFITM3*) is a common overregulated gene in all five datasets of colon cancer and is an immune-related protein involved in tumor transformation, with protein turnover controlled by autophagy (Friedlová et al., 2022). Y20 phosphorylation of IFITM3 hinders adaptor complex AP-2 recognizing its YEML motif, which is responsible for IFITM3 endocytosis (Chesarino et al., 2014). In colon cancer, adaptor related protein complex 1 subunit mu 1 (AP1M1), subunit sigma 2 (AP1S2), and subunit sigma 3 (AP1S3) are common overregulated genes and might be related to the accumulation of IFITM3 protein on the cell surface (**Figure 7**). IFITM3 mutation within its endocytosis-promoting YXXФ motif converts IFITM3 into an enhancer of SARS-CoV-2 infection by promoting virus-cell fusion (Shi et al., 2021). Krüppel-like factor 4 (KLF4) is a common downregulated TF in cancer (**Figure 2**) that would inhibit IFITM3 (Li et al., 2011) and SOX4 expression (**Figure 4**). IFITM3 might be related to CCND1 and CDK4 upregulation (**Figure 7**) during cell growth (Gan et al., 2019). The IFITM family may also be mutated in gastric cancer, regulating the entry of viruses into host cells (Prelli Bozzo et al., 2021), activating IFI6 and FKBP10 (**Figure 8**) and leading to TF phosphorylation like STAT1 (DeDiego et al., 2019). IFITM1 overexpression is related to the migration and invasiveness of gastric cancer cells (Lee et al., 2012). IFITM2 promotes gastric cancer progression by promoting cell migration and invasion, and inducing EMT (Xu et al., 2017), and interacts with the SARS-CoV-2 S at the cell surface and virus-cell fusion in early endosomes (Prelli Bozzo et al., 2021). IFTIM3 promotes gastric cancer progression, metastasis, stemness, and chemoresistance through the crosstalk between signaling pathways (Chu et al., 2022) and the activation of integrin signaling pathways (Friedlová et al., 2022). IFITM2 and IFITM3 promote human coronavirus OC43 infection, as three distinct mutations in tyrosine phosphorylation convert IFITM1 and IFITM3 from inhibitors to enhancers of SARS-CoV and MERS-CoV spike protein-mediated entry, challenging the “rigid-membrane” hypothesis (**Table 1**) (Zhao et al., 2018).

HCV might be involved in the activation of important tumorigenic signaling pathways of lung (**Figure 6**) and colon cancer (**Figure 7**), interacting with several membrane receptors (**Table 2**). HCV upregulates mRNA and protein expression levels of SLC3A2 through NS3/4A-mediated oxidative stress, as well as SLC3A2/LAT1 complex levels, contributing to HCV-mediated pathogenesis (Nguyen et al., 2018). Solute carrier family 3 member 2 (SLC3A2) can associate with integrin-β chains like Integrin beta 4 (ITGB4) in lung cancer, thereby influencing integrin signaling, cell survival, and cell migration (Fort et al., 2007). In lung cancer, protein kinase membrane-associated tyrosine/threonine 1 (PKMYT1) might be related to the activation of the MAPK signaling pathway and 4EBP1 phosphorylation (**Figure 6**) (Fuchs and Bode, 2005), promoting cell proliferation and apoptosis resistance (Zhang et al., 2020). ITGB4 is a heterodimer that is a non-covalently associated transmembrane glycoprotein receptor that forms complexes that vary in their ligand-binding specificities. It is an important component of the ECM that affects cell adhesion, migration, invasion, proliferation, and apoptosis during viral infection, and its phosphorylation at Y1510 is involved in the regulation of the MAPK-MEK1-ERK1/2 signaling pathway (Meng et al., 2020). In lung cancer, ITGB4 might be related to leucine-rich repeat-containing protein 15 (LRRC15), which is involved in cell-cell and cell-matrix interactions and overexpressed in mesenchymal-derived tumors (Ray et al., 2022), exerting a metastatic invasion role in lung cancer (Ruan et al., 2022). *ITGB4* and urokinase-type plasminogen activator (uPA) encoded by *PLAU*, two common overregulated genes in lung cancer, promote angiogenesis via ERK1/2 phosphorylation, leading to cell growth (LaRusch et al., 2010; Breuss and Uhrin, 2012). The overexpression of eukaryotic translation initiation factor 4E (eIF4E)-binding protein 1 (4EBP1) in NSCLC patients has been related to a lower survival (Tang et al., 2022). 4EBP1 is a usual phosphorylation target of the mTOR and MAPK-ERK signaling pathways (**Figure 6**), with HPV-E6 as intermediary, causing its release from eIF4E to allow cap-dependent translation, protein synthesis, and cell growth to meet the increased metabolic demand (She et al., 2010). Low-density lipoprotein receptor-related (LDLR) protein 8 (*LRP8*) is a common overexpressed gene in lung cancer tissues and cell lines and is correlated with poor clinicopathological characteristics and prognosis by modulating the Wnt signaling pathway (Fang et al., 2022). LDLRs are highly conserved receptors for multiple alphaviruses, which infect vertebrate species and insect vectors separated by hundreds of millions of years of evolutionary history (Clark et al., 2022). HCV core protein directly binds to STAT1 to induce its hetero- or homodimerization, resulting in HCV resistance to IFN therapy (Anjum et al., 2013). HCV-1b core protein also induces miR-93-5p upregulation and inhibits the IFN signaling pathway by directly targeting IFNAR1, whereas the miR-93-5p-IFNAR1 axis regulates STAT1 phosphorylation, which plays a crucial role in cancer development (He et al., 2018). The multi-step process of HCV entry might be facilitated by various host factors, including the tight junction protein claudin-1 (CLDN1), which is required for efficient HCV virion accumulation at the tight junction from the basolateral membrane (So et al., 2023). BID contains a specific cleavage site recognized by HCV NS3/NS4A proteases, downstream apoptotic molecules of the mitochondrial pathway (Hsu et al., 2003). Chemokine (C-X-C motif) ligand (CXCL)10 belongs to the ELR-CXC family and is a pro-inflammatory cytokine secreted upon IFN-γ stimulation by different cell types. It is involved in a wide variety of processes such as chemotaxis, differentiation, innate defense following viral infection with the activation of peripheral immune cells, and the regulation of cell growth (Liu et al., 2011), and is induced by HCV NS3/4A (Schaefer et al., 2011). HCV core is the first synthesized protein upon viral infection, regulates viral and cell expression, induces tumorigenesis, modulates apoptosis, and suppresses host immunity (Ray and Ray, 2001). HCV core protein has a pro-proliferative role through the increase of c-myc stability (Park et al., 2011). IFN-γ production and T-cell responses are negatively regulated by suppressors of cytokine signaling (SOCS) family members SOCS1 and SOCS3 through the inhibition of the Jak-STAT pathway (Bode et al., 2003). HCV core protein inhibits T-cell responses by interacting with gC1qR (complement component 1 Q subcomponent-binding protein, mitochondrial [C1QBP] or hyaluronan-binding protein 1 [HABP1]), and SOCS1/3, suppressing STAT1/3 (Kittlesen et al., 2000). In colon cancer, *HABP3* is a common overregulated gene that might be involved in the interaction with SOCS3 and the expression of IFN-stimulated genes (**Figure 7**). HCV NS5A confers innate immune evasion by interacting with 2′,5′-oligoadenylate synthetase (2′,5′-OAS) and inhibiting IFN antiviral activity (Taguchi et al., 2004). Human monoclonal transbodies that interfere with HCV NS5A activities have led to HCV replication inhibition and host immunity restoration (Glab-ampai et al., 2017).

*Entamoeba histolytica* trophozoites (EHT) might be involved in the activation of important tumorigenic signaling pathways in colon (**Figure 7**) and gastric cancer (**Figure 8**), interacting with several membrane receptors (**Table 2**). EHT causes substantial damage to the colonic epithelial cells that detach from the substrate to eventually be phagocytosed by the parasite (Cornick and Chadee, 2017), causing amoebiasis in 50 million people and killing 100.000 individuals around the world (Shirley et al., 2020). EHT Fibronectin (FN)-binding molecule (EhFNR) is like human β1 integrin (Talamás-Rohana et al., 1998) and is involved in adhesion, migration, and the invasion process, as well as the mobilization of the receptor molecule from internal vesicles to the plasma membrane, playing an important role during tumor development (Hernández-Ramírez et al., 2007). FN is a major host ECM component that induces actin remodeling in the parasite in a RAB21-dependent manner, forming invadosomes that promote the chemotactic migration of the metastatic cancer cells and non-transformed cells by remodeling the ECM (Emmanuel et al., 2015). RAB31 may be involved in the formation of invadosomes in colon cancer, promoting actin dot formation under an FN-induced signal in EHT, invasion, and pathogen virulence (**Figure 7**). The interaction with enteropathogenic *Escherichia coli* could modify the virulence of EH to cause amebiasis, resulting in a marked upregulation of EH cysteine proteinase (CP) virulence factors, which are critical in tumor pathogenesis and progression (Fernández-López et al., 2019). The overexpression of the EHT cysteine protease EhCP112 provokes major epithelial injury, increasing intestinal epithelial permeability, likely due to apical erosion and claudin-1 and claudin-2 degradation (Cuellar et al., 2017). The key EHT virulence factor that elicits the fast release of mucin by goblet cells as cysteine protease 5 (EhCP5) couples with goblet cell αvβ3 receptors and degrades the colonic mucus layer at the site of invasion (Cornick et al., 2016), suggesting that integrin-α9 (ITGA9) might be related to amoebiasis infection. ITGA9 is expressed in colonic glandular epithelial cells at the fetal stage and in colon adenocarcinoma, but not in normal adults (Xu et al., 2021b). ACTN1 may positively interact with ITGA9 to promote proliferation, invasion, and EMT in colon cancer (Wang et al., 2023).

The initial epithelial damage produced by EHT is characterized by the opening of tight junctions, followed by a dramatic drop in transepithelial electrical resistance with the participation of EhCPADH complex that affects claudin-1 and occludin (Betanzos et al., 2013), and damages adherens junctions and desmosomes (Hernández-Nava et al., 2017). EhFNR may also be involved in adhesion, migration, and the invasion process, as well as the mobilization of the receptor molecule from internal vesicles to the plasma membrane, playing an important role during gastric tumor development (Hernández-Ramírez et al., 2007). RAB31 may also be involved in the formation of invadosomes in gastric cancer by promoting actin dot formation under the fibronectin-induced signal in EHT and thus playing an important role during invasion and modulating pathogen virulence (**Figure 8**). The interaction with enteropathogenic *E. coli* could modify the virulence of EH to cause amebiasis, resulting in a marked upregulation of EH CP during tumor pathogenesis and progression (Fernández-López et al., 2019). Integrin subunit alpha 5 (ITGA5) and integrin subunit beta 5 (ITGB5) may form a heterodimer in gastric cancer that positively interacts with ACTN1 (**Figure 8**) to promote proliferation, invasion, and EMT (Wang et al., 2023). Both integrins participate in the integrin-mediated signaling pathway, viral entry into the host, HPV infection, the phagosome, focal adhesion, ECM-receptor interaction, and the cell junction (**Supplementary Table 3**). ITGA5 participates in the positive regulation of cell migration, along with CLDN1 and Ephrin type-B receptor 2 (EPHB2), and angiogenesis, along with EPHB2, TNFAIP2, and TNFRSF12A. ITGB5 expression contributes to a poor prognosis and is significantly associated with ECM organization, cell-substrate adhesion, focal adhesion, ECM-receptor interaction, and the phagosome (Liu et al., 2021a). The key virulence factor in live EHT may also elicit the fast release of mucin by goblet cells as EhCP5 couples with goblet cell αvβ3 receptors and degrades the colonic mucus layer at the site of invasion (Cornick et al., 2016), suggesting that ITGA5, ITGB5, and integrin subunit beta like 1 (ITGBL1) might be related to amoebiasis infection in gastric cancer.

HBV might be involved in the activation of important tumorigenic signaling pathways of lung cancer (**Figure 6**), interacting with several membrane receptors (**Table 2**). HBV is a small single-strand circular DNA virus that may be able to attach to heparan sulfate (HS) molecules and cell-free HS or SDC1, a transmembrane (type I) HS proteoglycan (HSPG), which occurs within clusters of integrins of the extracellular matrix (ECM) (Shi et al., 2013), which are receptors encoded by a common DEG in lung cancer (**Supplementary Table 1**). HBV may also attach to members of the solute carrier family (Somiya et al., 2016), such as *SLC7A5*, another common overregulated gene in lung cancer (**Supplementary Table 1**). The interaction between SDC1 and HMMR, the hyaluronan-mediated motility receptor overregulated in lung cancer, is related to tumor cell motility and differentiation (Yeh et al., 2018), as well as the pathological stage, T classification, lymph node metastasis, and distant metastasis (Li et al., 2021). The interaction of HMMR with SLC7A11 activates ferroptosis, enhances the cytotoxic effect of CD8 +T cells, and regulates the tumor immune microenvironment (Shan et al., 2023), suggesting an interaction with SLC7A5 in lung cancer. SLC7A5 or large amino acid transporter 1 (LAT1) is a heterodimeric transmembrane protein complex that catalyzes amino acid transport. It belongs to the SLC7-APC (amino acid-polyamine-organocation) superfamily (Scalise et al., 2018), participates in the immunosuppressive lung tumor microenvironment, and is associated with a low response to immunotherapy (Liu et al., 2022), high cancer stem cell (CSC) activity, and shorter overall survival (Liu et al., 2021c). Solute carrier family 3 member 2 (SLC3A2), or CD98hc, encodes another subunit of heterodimeric amino acid transporter that is overregulated in six lung cancer datasets and establishes a heterodimeric transmembrane protein complex with SLC7A5 to catalyze amino acid transport (Chiduza et al., 2019). HBV has evolved strategies to counter Toll-like receptor responses by suppressing their expression, regulating downstream signaling pathways related to adaptive immunity, and facilitating viral persistence (Du et al., 2022), like the non-canonical p38 mitogen-activated protein kinase (MAPK13) pathways, and probably the following phosphorylation and activation of DLX5 (**Figure 6**). HBV X protein (HBx) affects transcription and HBV replication, and it seems to be implicated in the regulation of BIRC5, cyclin E, and CDK4, and induces p16 hypermethylation, retinoblastoma phosphorylation, and E2F and DNMT1 activity (Tavakolian et al., 2020).

*Salmonella enterica* serovar Typhimurium (SEST) might be involved in the activation of important tumorigenic signaling pathways in gastric cancer (**Figure 8**), interacting with membrane receptors (**Table 2**). SEST resides in a membrane-bound compartment called the *Salmonella*-containing vacuole (SCV), which interacts with early endosomes to acquire a subset of late endosomal/lysosomal proteins through four characterized regulators of endocytic recycling, present on the SCV after invasion (Smith et al., 2005), from where they deliver effector proteins to the host cell via the *Salmonella* pathogenesis island 2 (SPI-2) type III secretion system (T3SS), inhibiting the process of antigen presentation by mature MHCII molecules (Azimi et al., 2020). SPI-5 encodes Salmonella outer protein B, which is involved in neutrophil recurrence (Wallis and Galyov, 2000), the interaction with the chloride channel, ion balance in the host cell, the management of SVC to inhibit lysosome vacuole fusion (Jantsch et al., 2011), and the returning of Rab5 to the SCV, causing the aggregation of phosphatidylinositide-3-phosphate (Mallo et al., 2008). ARF6 and Rab4 associate immediately but their presence diminishes 60 min post-infection, whereas syntaxin13 association peaks at 60 min to regulate the recycling of MHC class I. RAB11 association also peaks at 60 min to regulate the recycling of CD44 (Smith et al., 2005). *RAB31* is a common overregulated gene in gastric cancer and it might be able to distinguish the intracellular vacuole of human-adapted and broad-host SEST, which may have implications for the understanding of the marked differences between SEST’s biology and the fine-tuning of T3SSs activity to adapt their function to the unique requirements of each SEST because differences in a single type III secretion effector protein result in fundamental changes to *Salmonella*’s intracellular niche (Spanò et al., 2011). Annexin A2 (phosphatidylinositol (4,5)-bisphosphate binding protein), p11 (S100 calcium binding protein A10 (S100A10)), and AHNAK nucleoprotein are required for the T3SS-mediated *Salmonella* invasion of cultured epithelial cells, and T3SS effector SopB is required for the recruitment of AnxA2 and AHNAK to *Salmonella* invasion sites; *Salmonella* can then intersect the host cell actin pathway via AnxA2 (Jolly et al., 2014). In gastric cancer, S100A10 and AHNAK2 are common DEGs that might be involved in *Salmonella* infection; however, annexin A2 is not (**Supplementary Table 3**), suggesting that the pathway might be actin independent or another protein is recruited to actin assembly sites at cellular membranes (**Figure 8**).

The study of the specific mechanisms by which the TRN and microbiome networks related to viral, bacterial, fungal, and parasite infections influence cancer establishment and progression in the oral-gut-lung axis will continue with: (1) single-cell transcriptomics and epigenomics in circulating extracellular vesicles (host-cells and microbially derived extracellular vesicles) and tissue tumoral cells in gastric, colon, and lung cancer patients to validate the TRN and analyze its relationship with the microbiome network; (2) proteomic and metabolomic analyses in circulating extracellular vesicles (host-cells and microbially derived extracellular vesicles) and tissue tumoral cells in gastric, colon, and lung cancer, in the same cohort, to study the formation of protein-protein interaction networks by TFs and transcription co-factors, the binding of gene expression regulatory complexes to their specific target genes, and the posttranslational regulation mediated by these regulatory complexes; (3) metaproteomic and metabolomic analyses of the microbiome network, which can activate or repress certain signal transduction pathways involved in inflammatory and tumorigenic processes; (4) multiomic analyses of inflammatory diseases, such as periodontitis, pulmonary arterial hypertension, and inflammatory bowel disease, in the development of tumorigenic processes in our population; (5) the development of three-dimensional cell organoid models of healthy individuals and cancer patients to study the response to microbiome and epidrugs controlling regulatory and epigenomics mechanisms related to the transcriptional and posttranslational regulation of host-microbiome TF networks in the treatment of tumorigenic processes within the framework of personalized medicine; and (6) exploring exosomes as new generation vehicles for cancer treatment, drug delivery, the control of signaling pathways, and genomic expression through the regulation of transcriptomic and epigenomic mechanisms. All steps are crucial for developing targeted advanced therapies against lung and gut cancer.

## 5 Conclusion

The global transcriptomic network analysis of gut and lung cancer highlights the impact of five TFs (SOX4, TCF3, TEAD4, ETV4, and FOXM1) that might be related to stem cell programing and cancer progression through the regulation of the expression of an important number of common deregulated genes, such as cancer-cell membrane receptors that interact with several microorganisms, including HTLV-1, HPV, EBV, and SARS−CoV−2. The regulatory function of SOX4, FOXM1, and ETV4, over other key TFs (**Figure 3**) and common DEGs, was highlighted in lung cancer, establishing key co-regulatory complexes in NSCLC and SCLC. The regulatory function of TEAD4, TCF3, ETV4, SOX4, and FOXM1, over other key TFs (**Figure 4**) and common DEGs, was highlighted in colon cancer, establishing key co-regulatory complexes. The regulatory function of TEAD4, ETV4, PRRX1, and FOXM1, over other key TFs (**Figure 5**) and common DEGs, was highlighted in gastric cancer, establishing key co-regulatory complexes. ETV4 and FOXM1 are the two common overregulated TFs in the three types of cancer and are important in the regulation of other key TFs and DEGs, as well as the formation of co-regulatory complexes, during the tumorigenic process in the gut-lung axis. KLF4 and ZBTB16 are the two common downregulated TFs in the three types of cancer that might control key overregulated TFs and DEGs, and therefore, their expression must be controlled by key overregulated TFs during the tumorigenic process. There are specific overregulated cancer-cell membrane receptors crucial for the interaction with HTVL-1, HPV, EBV, and SARS−CoV−2 in every type of cancer, which are regulated by the key TFs (**Table 2**) and might be involved in the regulation of the MAPK signaling pathway (**Figure 6**), the non-canonical Wnt signaling pathway (**Figure 7**), and the regulation of the IFN signaling pathway (**Figure 8**) during the lung and gut tumorigenic process.

Our global transcriptomic analysis suggests a complex crosstalk between the microbiome and cancer TRN, as shown by the co-expression of common DEGs that codify for membrane receptors and TFs, and consequently, the possible participation in the same biological process, along with all experimental studies that have demonstrated that the interaction of the microbiome and specific receptors identified as common DEGs in cancer might be able to activate signaling pathways that regulate gene expression. The actual evidence in the field highlighted receptors that might be involved in the crosstalk between the microbiome network and the host cell TRN in a spatial, temporal, and sequential manner during the tumorigenic process in the gut-lung axis. Additionally, the evidence identified signaling pathways activated downstream that might be implicated in the regulation of the host cancer cell TRN, the regulation of gene expression during the establishment and progression of cancer, and in turn in the regulation of the communication with the microbiome network.

The regulatory function of key TFs over all common DEGs must be validated experimentally to fully understand how they are involved in the interaction of host cancer cells and microbiome gene expression networks that might be able to unlock cell phenotypic plasticity for the acquisition of the hallmarks of cancer in the gut-lung axis. All our findings must be experimentally validated with proper methodologies to specifically prove how, when, and where: 1) the microbiome network is interacting with the membrane receptors of cancer-related cells; 2) the host-microbiome interaction activates the signaling pathways related to gene expression; and 3) the cancer TRN is regulating the crosstalk with the microbiome network. *In vitro* studies of single and multiple microorganism infection are necessary to gain insight into general entry mechanisms and the activation and/or silencing of specific signaling pathways, as are studies involving patient cohorts that aim to identify the clinical relevance of molecular host-virus interactions, which are essential in the development of novel diagnostic and treatment approaches that control the formation of microorganism-host protein complexes. Then, it will be necessary to identify which signaling pathway is regulating the expression of which TFs and which DEGs are being controlled by which TFs, and highlighting between those, the genes that codify for membrane receptors that interact with the microbiome network; only then will the regulation circle of the tumorigenic processes between the microbiome and cancer TRNs be fully complete.

## Data Availability

The original contributions presented in the study are included in the article/**Supplementary Material**. Further inquiries can be directed to the corresponding author.

## Supplementary material

Supplementary Methods 1 Bioinformatic pipeline.

Supplementary Table 1 Lung cancer common DEGs selection, annotation, and gene regulatory networks.

Supplementary Table 2 Colon cancer common DEGs selection, annotation, and gene regulatory networks.

Supplementary Table 3 Gastric cancer common DEGs selection, annotation, and gene regulatory networks.
