# Supplementary material for: Global transcriptomic network analysis of the crosstalk between microbiota and cancer-related cells in the oral-gut-lung axis
Source: Front Cell Infect Microbiol. 2024 Aug 20;14:1425388. doi: 10.3389/fcimb.2024.1425388 (PMC11368877; doi:10.3389/fcimb.2024.1425388)
Supplement: Supplementary Methods 1 — Bioinformatic pipeline. [file Table4.docx]

################################################################

# Preprocesing Expression data

# Differential expression analysis

library(DESeq2)

library (Limma)

# load counts table from GEO

urld <- "hTable_aps://www.ncbi.nlm.nih.gov/geo/download/?format=file&type=rnaseq_counts"

path <- paste(urld, "acc=GSECode", "file=groupsECode_raw_counts_GRCh38.p13_NCBI.tsv.gz", sep="&");

table <- as.matrix(data.table::fread(path, header=T, colClasses="integer"), rownames="GeneID")

# load gene annations

path_a <- paste(urld, "type=rnaseq_counts", "file=Human.GRCh38.p13.ann.tsv.gz", sep="&")

ann <- data.table::fread(path_a, header=T, quote="", stringroupsAsFactors=F, data.table=F)

rownames(ann) <- ann$GeneID

# sample selection

groupsms <- "XXXXX1111100000" #Depending of the structure of the dataset 1 means cancer samples, 0 control samples, X useful samples

sml <- strsplit(groupsms, split="")[[1]]

# filter out excluded samples (marked as "X")

sel <- which(sml != "X")

sml <- sml[sel]

table <- table[ ,sel]

# group membership for samples

groups <- factor(sml)

groups <- make.names(c("Cancer","Control"))

levels(groups) <- groups

sample_info <- data.frame(Group = groups, row.names = colnames(table))

# pre-filter low count genes

# keep genes with at least N counts > 10, where N = size of smallest group

keep <- rowSums( table >= 10 ) >= min(table(groups))

table <- table[keep, ]

ds <- DESeqDataSetFromMatrix(countData=table, colData=sample_info, design= ~Group)

ds <- DESeq(ds, test="Wald", sfType="poscount")

# extract results for top genes table

r <- results (ds, contrast=c("Group", groups[1], groups[2]), alpha=0.05, pAdjustMethod ="fdr")

Table_a <- r[order(r$padj)[1:250],]

Table_a <- merge(as.data.frame(Table_a), ann, by=0, sort=F)

Table_a <- subset(Table_a, select=c("GeneID","padj","pvalue","lfcSE","stat","log2FoldChange","baseMean","Symbol","Description"))

write.csv(Table_a, file="toptable_GSECode.csv", row.names=F, sep="\t")

#Quality controls

plotDispEsts(ds, main="groupsECode Dispersion Estimates")

# create histogram plot of p-values

hist(r$padj, breaks=seq(0, 1, length = 21), col = "grey", border = "white",

xlab = "", ylab = "", main = "groupsECode Frequencies of padj-values")

# volcano plot

old.pal <- paleTable_ae(c("#00BFFF", "#FF3030")) # low-hi colors

par(mar=c(4,4,2,1), cex.main=1.5)

plot(r$log2FoldChange, -log10(r$padj), main=paste(groups[1], "vs", groups[2]),

xlab="log2FC", ylab="-log10(Padj)", pch=20, cex=0.5)

with(subset(r, padj<0.05 & abs(log2FoldChange) >= 0),

points(log2FoldChange, -log10(padj), pch=20, col=(sign(log2FoldChange) + 3)/2, cex=1))

legend("boTable_aomleft", title=paste("Padj<", 0.05, sep=""), legend=c("down", "up"), pch=20,col=1:2)

# MD plot

par(mar=c(4,4,2,1), cex.main=1.5)

plot(log10(r$baseMean), r$log2FoldChange, main=paste(groups[1], "vs", groups[2]),

xlab="log10(mean of normalized counts)", ylab="log2FoldChange", pch=20, cex=0.5)

with(subset(r, padj<0.05 & abs(log2FoldChange) >= 0),

points(log10(baseMean), log2FoldChange, pch=20, col=(sign(log2FoldChange) + 3)/2, cex=1))

legend("boTable_aomleft", title=paste("Padj<", 0.05, sep=""), legend=c("down", "up"), pch=20,col=1:2)

abline(h=0)

paleTable_ae(old.pal) # restore paleTable_ae

################################################################

# General expression data visualization

dat <- log10(counts(ds, normalized = T) + 1) # extract normalized counts

# box-and-whisker plot

lbl <- "log10(raw counts + 1)"

ord <- order(groups) # order samples by group

paleTable_ae(c("#1B9E77", "#7570B3", "#E7298A", "#E6AB02", "#D95F02",

"#66A61E", "#A6761D", "#B32424", "#B324B3", "#666666"))

par(mar=c(7,4,2,1))

boxplot(dat[,ord], boxwex=0.6, notch=T, main="groupsECode", ylab="lg(norm.counts)", outline=F, las=2, col=groups[ord])

legend("topleft", groups, fill=paleTable_ae(), bty="n")

# UMAP plot (multi-dimensional scaling)

library(umap)

dat <- dat[!duplicated(dat), ] # first remove duplicates

par(mar=c(3,3,2,6), xpd=TRUE, cex.main=1.5)

ump <- umap(t(dat), n_neighbors = 5, random_state = 123)

plot(ump$layout, main="UMAP plot, nbrs=5", xlab="", ylab="", col=groups, pch=20, cex=1.5)

legend("topright", inset=c(-0.15,0), legend=groups, pch=20,

col=1:length(groups), title="Group", pt.cex=1.5)

# Get the normalized count matrix

normalized_counts <- counts(ds, normalized = TRUE)

write.table(normalized_counts, file = "normalized_counts.txt", sep=",", quote = FALSE)

################################################

######Affymetrix processing

# Load necessary libraries

library(GEOquery)

library(limma)

# Download data from CodeGSE dataset

gse <- getGEO("CodeGSE")

# Access normalized data

data <- exprs(gse[[1]])

# Access sample labels (cancer vs normal)

sample_info <- pData(phenoData(gse[[1]]))

conditions <- sample_info$characteristics_ch1.0.0

# Check unique conditions

table(conditions)

# Define the design matrix for contrast

design <- model.matrix(~0 + conditions)

# Assign columns to cancer and normal conditions

colnames(design) <- levels(factor(conditions))

# Fit the linear model

fit <- lmFit(data, design)

# Perform contrast

contrast.matrix <- makeContrasts(conditionsCancer - conditionsNormal, levels = design)

# Fit the contrast model

fit.contrast <- contrasts.fit(fit, contrast.matrix)

# Get contrast statistics

fit.contrast <- eBayes(fit.contrast)

# Extract differentially expressed genes

topTable <- topTable(fit.contrast, coef = 1, number = Inf)

# Save the result table to a CSV file

write.csv(topTable, file = "toptable_CodeGSE.csv", row.names = TRUE)

# Normalize counts and save to a text file

normalized_counts <- normalizeBetweenArrays(data, method = "quantile")

write.table(normalized_counts, file = "normalized_counts.txt", sep = "\t", quote = FALSE)

##################################################

########Calculating the matrix of expresión of DEG

# Load libraries

library(GEOquery)

library(limma)

# Set working directory

setwd("C:/Colon/CodeGSE")

# Load normalized expression matrix of CodeGSE

exprs_data <- read.table("normalized_counts.txt", header = TRUE, sep = "\t", stringroupsAsFactors = FALSE)

# Load the CodeGSE.top.table file (TSV file)

top_table <- read.table("toptable_groupGSECode.csv", header = TRUE, sep = "\t", stringroupsAsFactors = FALSE)

# Select differentially expressed genes

selected_genes <- top_table[top_table$ID %in% rownames(exprs_data) & (top_table$logFC >= 0.584 | top_table$logFC <= -0.584) & top_table$adj.P.Val < 0.05, c("Gene.symbol", "ID")]

# Filter expression matrix with selected genes using the "ID" column

filtered_exprs_data <- exprs_data[selected_genes$ID, ]

# Add the "Gene.symbol" column at the beginning

filtered_exprs_data <- cbind(Gene.symbol = selected_genes$Gene.symbol, filtered_exprs_data)

# Write the CSV file with differentially expressed genes

write.csv(filtered_exprs_data, file = "CodeGSE_DEG_expresion.csv", row.names = TRUE)

# Print a message indicating the process is complete

cat("Process completed. Files CodeGSE_Matriz_expresion.csv and CodeGSE_DEG_expresion.csv created in", getwd(), "\n")

#Expression Matrix construction with common overregulated genes

setwd("C:/Users/Desktop/Cancer...")

library(dplyr)

eset1<-read.table("GSE...DEG_Normalized.txt",h=T)

eset1<- eset1 %>% distinct(Symbol, .keep_all = TRUE)

dim(eset1)

names(eset1)

head(eset1)

lista<-read.table("GENESCW.txt")

lista<-as.character(lista$V1)

lista<-unique(lista)

length(lista)

class(lista)

todos<-eset1$Symbol

ind<-which(todos%in%lista)

length(ind)

E=eset1[ind,]

dim(E)

names(E)

head(E)

genes<-E$Symbol

Em<-as.matrix(E[,-1])

dim(Em)

rownames(Em)<-genes

#Transcriptional regulatory network construction

library(RTN)

TFlist <-c("X1","X2",...)

RTN <- tni.constructor(expData = Em, regulatoryElements = TFlist)

rtni <- tni.permutation(RTN, nPermutations = 100)

rtni <- tni.bootstrap(rtni)

rtni <- tni.dpi.filter(rtni)

tni.regulon.summary(rtni)

tni.regulon.summary(rtni, regulatoryElements = "X1")

tni.regulon.summary(rtni, regulatoryElements = "X2")

regulons <- tni.get(rtni, what = "regulons.and.mode", idkey = "ID")

head(regulons$SOX4)

head(regulons$SOX17)

g <- tni.graph(rtni, regulatoryElements = c("X1","X2",...))

library(RedeR)

rdp <- RedPort()

calld(rdp)

addGraph(rdp, g, layout=NULL)

addLegend.color(rdp, g, type="edge")

addLegend.shape(rdp, g)

relax(rdp, ps = TRUE)

#Transcriptional coregulatory network construction

library("CoRegNet")

TFlist <-c(""X1","X2",...)

grn = hLICORN(Em, TFlist=TFlist)

influence = regulatorInfluence(grn,Em)

coregs = coregulators(grn)

coregs

write.table(coregs,"GSE...COREGSUPSREGS.txt")

display(grn,Em,influence)

print(grn)

Rnet <- coregnetToDataframe(grn)

write.table(Rnet,"GSE...TFSGRN.txt")
